# Tyrosyl-DNA phosphodiesterase 2 (Tdp2) repairs DNA-protein crosslinks and protects against double strand breaks *in vivo*

**DOI:** 10.3389/fcell.2024.1394531

**Published:** 2024-08-20

**Authors:** Ivan Anticevic, Cecile Otten, Marta Popovic

**Affiliations:** DNA Damage Group, Laboratory for Molecular Ecotoxicology, Department for Marine and Environmental Research, Institute Ruder Boskovic, Zagreb, Croatia

**Keywords:** DNA repair, DNA-protein crosslinks, Tyrosyl-DNA phosphodiesterase 2 (TDP2), zebrafish, Topoisomerase 2, Ku80, Tyrosyl-DNA phosphodiesterase 1 (TDP1)

## Abstract

DNA-protein crosslinks pose a significant challenge to genome stability and cell viability. Efficient repair of DPCs is crucial for preserving genomic integrity and preventing the accumulation of DNA damage. Despite recent advances in our understanding of DPC repair, many aspects of this process, especially at the organismal level, remain elusive. In this study, we used zebrafish as a model organism to investigate the role of TDP2 (Tyrosyl-DNA phosphodiesterase 2) in DPC repair. We characterized the two *tdp2* orthologs in zebrafish using phylogenetic, syntenic and expression analysis and investigated the phenotypic consequences of *tdp2* silencing in zebrafish embryos. We then quantified the effects of *tdp2a* and *tdp2b* silencing on cellular DPC levels and DSB accumulation in zebrafish embryos. Our findings revealed that *tdp2b* is the main ortholog during embryonic development, while both orthologs are ubiquitously present in adult tissues. Notably, the *tdp2b* ortholog is phylogenetically closer to human TDP2. Silencing of *tdp2b*, but not *tdp2a*, resulted in the loss of Tdp2 activity in zebrafish embryos, accompanied by the accumulation of DPCs and DSBs. Our findings contribute to a more comprehensive understanding of DPC repair at the organismal level and underscore the significance of TDP2 in maintaining genome stability.

## Introduction

DNA-protein crosslinks (DPCs) are irreversible covalent linkages between DNA and proteins that can arise from endogenous cellular processes or exposure to genotoxic agents (Swenberg et al., 2011). Crosslinked proteins block all DNA transactions including replication, transcription, and repair (Vaz et al., 2017; Fielden et al., 2018; Ruggiano and Ramadan, 2021). If left unrepaired, DPCs cause genomic instability and/or cell death which in turns can lead to the development of diseases, including cancer, neurodegenerative disorders, and aging-related conditions (Takashima et al., 2002; Hirano et al., 2007; Lessel et al., 2014; Zagnoli-Vieira et al., 2018; Scott et al., 2019). Efficient repair of DPCs involves a complex interplay of distinct pathways. The recent discovery of a proteolytic pathway revealed the role of the proteases Wss1 (Weak suppressor of SMT3 protein 1) in yeast (Stingele et al., 2014) and SPRTN (SprT-like N-terminal domain) in metazoans (Lopez-Mosqueda et al., 2016; Stingele et al., 2016; Vaz et al., 2016; Mórocz et al., 2017), which initiate DPC repair by directly degrading crosslinked proteins. Recently, other proteases such as ACRC/GCNA (Acidic repeat-containing protein/Germ cell nuclear acidic peptidase), FAM111A (Family with sequence similarity 111 member A), DDI1 and 2 (DNA-damage inducible 1 and 2), and the proteasome have also been associated with DPC repair (Larsen et al., 2019; Bhargava et al., 2020; Hoffmann et al., 2020; Kojima et al., 2020; Serbyn et al., 2020; Ruggiano and Ramadan, 2021; Otten et al., 2023). An alternative mechanism to proteolysis is a nuclease-mediated repair, in which the crosslinked protein is removed along with the excised DNA. The NER (Nucleotide Excision Repair) pathway has been shown to remove smaller DPCs of up to 12–14 kDa *in vitro* and in bacterial cells (Nakano et al., 2007), and up to 38 kDa in human cells (Chesner and Campbell, 2018). Besides NER, it has been shown that MRE11 (Meiotic Recombination 11) can remove TOP2-DPCs (Deshpande et al., 2016; Hoa et al., 2016), while recently, APEX1 and APEX2 (Apurinic/Apyrimidinic Endodeoxyribonuclease) and FEN1 (Flap structure-specific endonuclease 1) have also been identified to play a role in this process (Álvarez-Quilón et al., 2020; Zhang et al., 2022; Sun et al., 2023).

In the proteolytic pathway, the specialized enzymes tyrosyl-DNA phosphodiesterase 1 (TDP1) and 2 (TDP2) play a crucial role in the direct reversal of covalent bonds between protein residues and DNA after protein debulking (Pommier et al., 2014; Kawale and Povirk, 2018). While TDP1 removes crosslinked peptides of topoisomerase 1 (TOP1), histone H3 and possibly other proteins (Anticevic et al., 2023), TDP2 plays a crucial role in the resolution of crosslinked peptides that remain after irreversible binding of topoisomerase 2 to DNA (TOP2-DPCs). This occurs during the catalytic cycle of TOP2, during which it generates transient DNA breaks to alleviate helical stress (Chen et al., 1984; Zeng et al., 2011). The trapping of TOP2 to DNA can occur due to exposure to aldehydes or reactive oxygen species during normal cellular functions (Shoulkamy et al., 2012; Tretyakova et al., 2015), or to anticancer drugs such as etoposide or doxorubicin, leading to persistent DNA damage and cytotoxicity (Nitiss, 2009; Wu et al., 2011). Therefore, TDP2 emerged as a potential target for anticancer therapy (Dexheimer et al., 2008) and TDP2 inhibitors are under development (Laev et al., 2016; Sun et al., 2020).

Apart from its role in DPC repair, TDP2 is also known as TTRAP or EAP II and is involved in several cellular processes, including NF- κβ signaling (Pype et al., 2000), MAPK-ERK signaling (Li et al., 2011), and HIV-1 integration (Zhang et al., 2009). Also, TDP2 plays a crucial role in non-homologous end-joining (NHEJ), a major DNA double-strand break repair pathway and contributes to error-free repair of TOP2-induced DSBs and protection against drug-induced mutagenesis (Gómez-Herreros et al., 2013).

Loss of TDP2 in mice leads to significant changes in genome-wide expression profiles, with over 100 genes downregulated in TDP2-deficient neurons compared to WT neurons (Gómez-Herreros et al., 2014). Approximately half of these genes are associated with the etiology of seizures/epilepsy, ataxia, and cognitive development. Indeed, specific mutations in *TDP2* have been linked to the human genetic disorder Spinocerebellar ataxia autosomal recessive 23 (SCAR23), which is characterized by intellectual disability, seizures, and ataxia (Zagnoli-Vieira et al., 2018; Errichiello et al., 2020).

While much is known about TDP2 and its involvement in DPC repair from *in vitro* studies and cellular models (Zeng et al., 2011; Marchand et al., 2014; Schellenberg et al., 2017; Lee et al., 2018), its role in DPC repair at the organismal level has not yet been investigated. Phenotypes in adult mice with impaired TDP2 have shown weight loss due to intestinal damage and increased toxicity in lymphoid tissue after etoposide administration (Gómez-Herreros et al., 2013), but DPC repair has not been studied in TDP2-deficient mice or in cells derived from SCAR23 patients (Gómez-Herreros et al., 2013; Zagnoli-Vieira et al., 2018). Understanding the role of TDP2 in DPC repair in animal models is of great importance for the treatment of cancer and potentially for the treatment of neurological disorders associated with loss of TDP2 function. The TOP2 poison etoposide and its derivatives are already used in the treatment of systemic cancers and many solid tumors (Vann et al., 2021). However, there is a need for improvement due to side effects and dose-dependent toxicity. Therefore, the development of TDP2 inhibitors that could be used in synergy with TOP2 poisons could improve current clinical treatments (Laev et al., 2016; Kankanala et al., 2019).

Our aim was to investigate the role of Tdp2 in DPC repair using zebrafish, a powerful model organism to study DNA repair and its effects on aging, cancer, and neurodegeneration (Zhao et al., 2015; Lin et al., 2016; Cayuela et al., 2019; Choi et al., 2021). Considering that the DNA repair pathways are 99% conserved between humans and zebrafish (Abugable et al., 2019), that physiological processes are very similar (Teame et al., 2019), and that significantly more samples can be analyzed compared to the mouse model, the zebrafish is ideally suited for studying the molecular mechanisms underlying the disease phenotypes. Other clear advantages over the mouse model are the easier genetic manipulation due to external fertilization and the optical transparency of the embryos, as well as the much higher fecundity which enables better statistical analysis of DPC levels in embryos and adults (Gemberling et al., 2013; Choi et al., 2021).

In this study, we characterized *tdp2a* and *tdp2b*, the two *tdp2* orthologs in zebrafish using phylogenetic, syntenic and expression analysis and investigated their role in DPC repair at the organismal level. Phylogenetic and domain analysis revealed that human TDP2 is more similar to the zebrafish *tdp2b* ortholog, while synteny showed duplicated gene environments. We further showed that *tdp2b* is more abundantly expressed in embryonic development compared to *tdp2a.* In adults, both orthologs are ubiquitously expressed across all examined tissues, with gender-specific gene expression observed in gonads where *tdp2a* is highly expressed in testes, while *tdp2b* is predominantly expressed in ovaries. In zebrafish embryos, we successfully optimized silencing of both *tdp2* orthologs using a morpholino approach, and restored Tdp2 function by overexpressing Tdp2b. Silencing of *tdp2b*, but not *tdp2a*, resulted in a substantial loss of Tdp2 activity, further supporting the dominant role of Tdp2b compared to Tdp2a. Tdp2b deficiency led to a significant accumulation of cellular DPCs and DSBs. Our findings underscore the critical role of *tdp2b* in maintaining genome stability during vertebrate embryonic development, offering valuable insights into DNA repair-related diseases that could lead to the development of novel strategies for addressing DNA damage-related disorders and improving chemotherapeutic approaches.

## Materials and methods

### Phylogenetic, syntenic and domain analyses

Phylogenetic analysis was performed with the Maximum Likelihood method in SeaView software (Gouy et al., 2010) using the PhyML program with the following parameters: LG model, 8 rates of categories, tree searching operation best of NNI&SPR (Nearest Neighbor Interchange and Subtree Pruning and Regrafting) (Guindon and Gascuel, 2003). Tree node confidence is expressed as Alrt values (Approximate likelihood-ratio test) on a scale of 0–1, where 1 represents the maximum node confidence (Anisimova and Gascuel, 2006). Protein sequences were retrieved from the National Center for Biotechnology Information (NCBI) database (Benson et al., 2013) using the blastp algorithm (Altschul et al., 1990) with human TDP2 as the query sequence, followed by alignment of the full-length protein sequences using the Multiple Alignment using Fast Fourier Transform (MAFFT) algorithm (Katoh et al., 2002). Alignment quality was assessed using the Guidance2 server and the score was 0.767980 which corresponds to high alignment quality. For comparison, a score above 0.5 is considered sufficient to use the alignment for tree building using the maximum likelihood method (Penn et al., 2010). Syntenic analysis of the *TDP2* gene was performed using Genomics, a browser for conserved synteny synchronized with genomes from the Ensembl database (Louis et al., 2013). Protein domain structures of human TDP2 and zebrafish Tdp2a and Tdp2b were visualized using IBS software (W. Liu et al., 2015).

### Zebrafish husbandry and exposure experiments

The zebrafish (*Danio rerio*) AB strain was obtained from the European Zebrafish Resource Centre (EZRC, Karlsruhe, Germany) and was maintained at a temperature of 28°C under a 14-h light and 10-h dark cycle as previously described (Aleström et al., 2020). *Tdp1* ^*−/−*^ mutants were created in our lab and characterized in Anticevic et al., 2023. Embryos were cultured in E3 media (5 mM NaCl, 0.17 mM KCl, 0.33 mM CaCl_2_, and 0.33 mM MgSO_4_) at 28°C until 2 days post-fertilization (dpf). Prior to experiments, embryos were manually dechorionated and, if required, treated with 10 mM formaldehyde for 30 min (FA, KEMIKA: 0633501) or with 50 μM etoposide (ETO, Thermo Scientific Chemicals: J63651) for 1 h at 28°C. All procedures followed ethical guidelines (EU Directive 86/609/EEC, Croatian Federal Act on Animal Protection) under project license HR-POK-023.

### RNA isolation and qPCR analysis from zebrafish tissue and embryos

RNA isolation from adult zebrafish tissue samples weighing up to 50 mg was performed using the Monarch Total RNA Miniprep Kit (NEB, T2040L). The tissue samples were homogenized using an Ultra Turrax T25 homogenizer at medium intensity for 60 s (13,500 rpm) followed by 5 min incubation with proteinase K (20 mg/mL) at 55°C. After centrifugation at 13,000 g for 2 min at room temperature, the supernatant was separated, and RNA purification was carried out following the manufacturer’s instructions. Five embryos per condition at different developmental stages were collected, including 6 h post-fertilization (6 hpf) and 1, 2, 3, 4, and 5 days post-fertilization (dpf), respectively. Two-day-old morphant embryos were collected after *tdp2a* or *tdp2b* gene silencing to determine the *tdp2* expression levels after the respective gene silencing. Pools of five embryos were collected at 2 dpf to determine the expression of the injected mRNAs: *tdp2b*, *tdp2b* ^*D285A*^, *tdp2a*, and *HsTDP2*. For the extraction of RNA, samples were sonicated 3 × 5 s on ice, followed by proteinase K treatment according to manufacturer’s instructions using the Monarch Total RNA Miniprep Kit (NEB, T2040L). The isolated RNA was subsequently aliquoted and stored at −80°C. For reverse transcription, the ProtoScript II First Strand cDNA Synthesis Kit (NEB, E6560L) was used, following the manufacturer’s instructions. Total RNA from zebrafish tissues and embryos was added in a volume corresponding to 100–1000 ng of RNA, resulting in a concentration of 5–50 ng/μL of cDNA for subsequent expression analysis.

qPCR analysis was conducted using GoTaq qPCR mix (PROMEGA, A6001) (Table 1). The housekeeping gene *atp50* (*atp5po*, ATP synthase peripheral stalk subunit OSCP, Gene ID: 335191) was used as a reference gene for normalization. Quantification was performed using the Qgene method (Simon, 2003), and gene expression levels were reported as Mean Normalized Expression (MNE). MNE was calculated based on the primer efficiencies (E) and mean Ct values for both the housekeeping gene (Ct (HKG)) and the target gene (Ct (gene)), using the equation: $MNE=\;{E\left(HKG\right)}^{Ct\left(HKG\right)}/{E\left(gene\right)}^{Ct\left(gene\right)}\times 10^{6}$ as previously described (Popovic et al., 2010; Lončar et al., 2016; Mihaljevic et al., 2016).

**TABLE 1:** Primers for qPCR analysis of gene expression in adult zebrafish and embryos.

Primer	Sequence
tdp2a-F	5′-CAG​AGT​CTC​TCC​AAT​GTC​AAT​CCA-3′
tdp2a-R	5′-TGG​GTG​CAC​TTG​GTT​TCT​GT-3′
tdp2b-F	5′-ATG​GAT​TCA​GTC​TTC​GAT​GAG​G-3′
tdp2b-R	5′-CTG​TCA​AGT​CAA​TGC​AAT​CCG​C-3′
HsTDP2-F	5′- CCA​GTA​TAC​ATG​GGA​TAC​ACA​AAT​G -3′
HsTDP2-R	5′- TCT​GCT​GCT​GCT​CTG​AAA​AAT​A -3′
atp50-F	5′-CTT​GCA​GAG​CTG​AAA​GTG​GC-3′
atp50-R	5′-ACC​ACC​AAG​GAT​TGA​GGC​AT-3′

### Gene silencing of zebrafish *tdp2a* and *tdp2b* genes with morpholino oligonucleotides

The antisense morpholino oligonucleotides targeting *tdp2a* and *tdp2b* were designed and ordered from Genetools LLC (Nasevicius and Ekker, 2000). In particular, the *tdp2a* morpholino targets exon 3–intron 3 boundary and the *tdp2b* morpholino targets the 5′UTR to prevent splicing and translation, respectively (Table 2). Morpholinos were diluted in a 0.015% Phenol-red/300 mM KCl solution to obtain injection mixes containing 500 μM *tdp2a*MO, 300 μM *tdp2b*MO, or 500µM + 300 μM *tdp2a* + *tdp2b*MO; 1nL injection mix was injected into zebrafish embryos between the one and the four-cell stage.

**TABLE 2:** Morpholino antisense oligonucleotides used for gene silencing.

Morpholino name	Sequence
*tdp2a*MO	5′-TGC​GAT​CTT​TGA​CAT​ACC​TTC​CAG​A-3′
*tdp2b*MO	5′-TCA​CAG​TTT​AAT​ATA​ACG​GCG​GGC​T-3′

To verify the efficiency of the splice-blocking *tdp2a* morpholino, RNA was extracted from 2 dpf embryos, reverse-transcribed to cDNA as described above, and PCRs were performed on those cDNA samples to determine the effects of the morpholino on transcript splicing as previously described (Anticevic et al., 2023). The expected size of the amplicon on WT samples is 524 bp, using the primer pair shown in Table 3. To verify the efficiency of t*dp2b* morpholino, a functional assay was performed to quantify the enzymatic activity of Tdp2b to confirm gene silencing (Moulton, 2007).

**TABLE 3:** Oligonucleotides used for determining efficiency of morpholino silencing.

Oligonucleotide name	Sequence
*tdp2a*MO-F	5′-CAG​CGC​AAG​AAG​CAA​TCA​TC-3′
*tdp2a*MO-R	5′-CAG​AGA​TAC​CAT​CCG​GCA​AC-3′

### Transient Tdp2b overexpression in zebrafish embryos

To verify the specificity of the *tdp2b* morpholino, we performed rescue experiments in which we co-injected *tdp2b*MO and mRNA encoding full-length *tdp2b* coding sequence into one-cell stage embryos. Since the *tdp2b* morpholino targets the endogenous 5′UTR of *tdp2b*, it cannot bind to the mRNA rescue construct which has a different upstream sequence derived from the plasmid from which it was *in vitro* transcribed. DrTdp2b (NM001079703.1) coding sequence was amplified using Infusion primers (shown in Table 4) on cDNA derived from 6 hpf WT embryos. The PCR product was then cloned into the pCS2+HisMyc vector between the XhoI and XbaI restriction sites using the Infusion kit (Takara Bio USA, Inc.) (Rohr et al., 2006). The resulting plasmid was mutated using the primer shown in Table 4 to obtain a catalytically inactive Tdp2b^D285A^. Zebrafish DrTdp2a (ENSDART00000102212.5) coding sequence was amplified using infusion primers (Table 4) from cDNA derived from the intestine of adult male zebrafish and cloned into the pCS2+HisMyc vector. Human TDP2 (NM_016614.3) was obtained from Genscript and cloned into the pCS2+HisMyc using infusion primers (Table 4). All plasmids were linearized using the NotI restriction enzyme and *in vitro* transcribed using the HiScribe SP6 RNA kit (NEB, #E2070) in conjunction with the ARCA kit (NEB, #S1411) to cap the resulting RNAs which were then purified using the Monarch RNA cleanup kit (NEB, #T2040) for subsequent injections. For injection experiments, 1 nL of a solution of mRNA alone (250 ng/ul) or mRNA (250 ng/ul) with *tdp2b*MO (300 μM) in 300 μM KCl was injected between the 1- and 4-cell stage.

**TABLE 4:** Oligonucleotides used for cloning the rescue constructs.

Oligonucleotide name	Sequence
DrTdp2b-Inf-F	5′- AGA​GGA​TCT​GCT​CGA​GAT​GTC​TGC​TCT​GGA​GGA​ATC​C-3′
DrTdp2b-Inf-R	5′- TCA​CTA​TAG​TTC​TAG​ATC​ATG​TGT​TGA​AAG​TGC​AGT-3′
DrTdp2b-D285A	5′- TGT​CAT​TTT​TGC​AGG​CGC​CAC​AAA​TCT​CAG​AGA​CG -3′
HsTDP2-Inf-F	5′- AGA​GGA​TCT​GCT​CGA​GAT​GGA​GTT​GGG​GAG​TTG​CCT​G -3′
HsTDP2-Inf-R	5′- TCA​CTA​TAG​TTC​TAG​ATT​ACA​ATA​TTA​TAT​CTA​AGT​TGC​AC -3′
DrTdp2a-Inf-F	5′- AGA​GGA​TCT​GCT​CGA​GAT​GGA​TAA​CCC​ATC​CTG​TGT​ACA -3′
DrTdp2a-Inf-R	5′- TCA​CTA​TAG​TTC​TAG​ATC​AGT​CAG​TGA​CAC​ACT​GTT​CTT​CT -3′

### TDP2 activity assay

The Cy5-labeled substrate oligomer (100 pmol) (Table 5) was mixed with a 20-bp complementary oligonucleotide containing a 5′overhang in a volume of 33.3 μL. The sample was denatured at 95°C for 5 min and then reannealed by gradually reducing the temperature at a rate of 2°C/s for 5 s, followed by 0.1°C/s for 600 s using the gradient PCR (T100 Thermal Cycler, Biorad). This process generated a 3 μM double-stranded substrate oligomer with a 5′overhang, which is a model substrate for TDP2 (Ledesma et al., 2009).

**TABLE 5:** Oligonucleotides used for Tdp1 and Tdp2 activity assays.

Oligonucleotide name	Sequence	Modification	Source
SUBSTRATE (Tdp1 activity)	5′-GATCTAAAAGACT3-3′	3′-pY, 5′-Cy5	Midland Certified Reagent Company, TX, United States
SUBSTRATE (Tdp2 activity)	5′-CAT​CGT​TGC​CTA​CCA​T-3′	5′-pY, 3′-Cy5	Midland Certified Reagent Company, TX, United States
COMPLEMENTARY	5′-GCA​TGA​TGG​TAG​GCA​ACG​ATG-3′	—	Macrogen (Europe)
COMPETITOR	5′-ATG​GTA​GGC​AAC​GAT​G-3′	—	Macrogen (Europe)

The Tdp2 activity assay was performed as previously described (Zagnoli-Vieira et al., 2018; Zaksauskaite et al., 2021) with few modifications. 2 dpf zebrafish embryos were deyolked in deyolking buffer (55 mM NaCl, 1.8 mM KCl and 1.25 mM NaHCO_3_, pH 8.5) and washed twice with deyolking wash buffer (110 mM NaCl, 3.5 mM KCl, 2.7 mM CaCl2 and 10 mM Tris-HCl, pH 8.5). The deyolked embryos were transferred in a solution containing 40 mM Tris/HCl pH 7.5, 100 mM NaCl, 0.1% Tween-20, 1 mM DTT, 1 mM PMSF, and protease inhibitors (leupeptin, aprotinin, chymostatin, pepstatin at a concentration of 1 μg/mL) and sonicated for 30 s using a probe sonicator with 3 μm peak-to-peak amplitude. Following sonication, the lysate was incubated for 30 min on ice and then centrifuged at 10,000 g for 5 min at 4°C. The supernatant, which contained proteins, was collected, and the protein concentration was determined using the Bradford assay (Bradford, 1976). Subsequently, 10 μg of the protein solution was mixed with 1 × Tdp2 activity assay buffer (50 mM Tris/HCl pH 8.0, 10 mM MgCl_2_, 80 mM KCl, 1 mM DTT, 0.01% Tween-20), the Cy5-labeled substrate oligomer (40 nM) and a competitor oligo (3 μM) (Table 5). The reaction was incubated for 1.5 h at 37°C, and stopped by the addition of 2x formamide loading buffer (80% (w/v) deionized formamide, 1 mg/mL xylene cyanole, 1 mg/mL bromophenol blue, and 10 mM EDTA (pH 8.0)) and boiled for 5 min at 95°C. The samples were then applied to a 20% polyacrylamide gel containing 8M urea, which had been pre-run for 1 h at 80 V. The gel was run for 2 h at 100 V to achieve optimal sample separation. The gel was visualized using the ChemiDoc MP imaging system (Bio-Rad, 1708280).

### TDP1 activity assay

The TDP1 activity assay optimized for detecting Tdp1 activity in zebrafish embryos was performed as described previously (Anticevic et al., 2023). In brief, 600 ng of embryo lysates were incubated with a Tdp1 oligonucleotide substrate (Table 5), containing a tyrosine at the 3′ end of the DNA and Cy5 at the 5′end. Active Tdp1 removes the tyrosine from the 3′ end, causing a shift in the size of the substrate. 2 dpf *tdp1* ^−/−^ mutants and WT embryos, with or without transient overexpression of *tdp2a* and *tdp2b* mRNA, were deyolked and homogenized for 10 s in 100 μL of lysis buffer (200 mM Hepes, 40 mM NaCl, 2 mM MgCl_2_, 0.5% Triton X-100 with protease inhibitors), followed by incubation on ice for 30 min. Next, the supernatant protein solution (600 ng) was incubated with 2.5 µM labeled oligonucleotide substrate in assay activity buffer (25 mM Hepes (pH 8.0), 130 mM KCl, and 1 mM dithiothreitol (DTT)) in a final reaction mixture of 10 μL. The reaction proceeded at 37°C for 1 h, after which loading buffer was added, and the mixture was boiled at 95°C for 5 min. All samples were loaded onto a pre-run 20% homemade urea gel and run at a constant voltage (120 V) for 2 h. The resulting oligonucleotide products were visualized using the ChemiDoc MP Imaging System to detect Cy5 fluorescence.

### DPC isolation from zebrafish embryos and analysis of total and specific DPCs

RADAR (rapid approach to DNA adduct recovery) assay is a well-known method for DPC isolation from cell models (Kiianitsa and Maizels, 2013; 2020). In this study, similar to our previous work (Anticevic et al., 2023), we employed a modified assay optimized for DPC isolation from zebrafish embryos, enabling better reproducibility and increased sensitivity. 2 dpf embryos were used for total DPC detection as previously described (Anticevic et al., 2023) and 1 dpf embryos were used for detection of specific DPCs (Top2, Ku80 and H3). In brief, embryos were collected and lysed using pre-warmed lysis buffer (6 M guanidinium thiocyanate (GTC), 10 mM Tris-HCl (pH 6.0), 20 mM EDTA, 4% Triton X100, 1% N-lauroylsarcosine sodium, and 1% β-mercaptoethanol), followed by incubation at 50°C for 5 min. The DNA with crosslinked proteins was precipitated by adding an equal volume of 98% ethanol, followed by centrifugation at 10,000 rcf for 10 min at 4°C. The resulting pellet was washed four times with wash buffer (20 mM Tris-HCl (pH 7.4), 1 mM EDTA, 50 mM NaCl, 50% EtOH) and dissolved in 8 mM NaOH (1 mL). To quantify the DNA content in the DPC samples, a 25 μL aliquot of each sample was treated with proteinase K (20 mg/mL) and quantified using the Pico Green assay according to the manufacturer’s instructions (Invitrogen, P7581). The DPC samples were normalized to the sample with the lowest DNA content and treated with DNAse (Millipore, E1014) for 1 h at 37°C. Subsequently, the DPC samples were snap-frozen in liquid nitrogen and subjected to overnight lyophilization using a FreeZone 2.5 lyophilizer (Labconco, United States). The lyophilized samples were dissolved in 50 μL SDS loading buffer containing 4 M urea, 62.5 mM Tris-HCl (pH 6.8), 1 mM EDTA, and 2% SDS.

To detect total DNA-protein crosslinks (DPCs), 250 ng of DNA-normalized DPCs isolated from 2 dpf zebrafish embryos were separated via SDS-PAGE electrophoresis. The DPC samples were mixed with 5x Laemmli buffer and 5% β-mercaptoethanol before being applied to homemade gradient 5%–18% polyacrylamide gels. Following electrophoresis, the resolved DPCs were visualized using silver staining (Sigma Aldrich, PROTSIL1). To detect specific DPCs, total cellular DPCs were isolated from 1 dpf embryos and applied to dot blot analysis using the Bio-Dot^®^ Microfiltration System (BioRad, 1703938). For Top2-DPCs, 1000 ng of DNA-normalized DPCs were transferred to a nitrocellulose membrane (GE10600002 Amersham™ Protran^®^) via vacuum aspiration. Similarly, 500 ng of DNA-normalized DPCs were used for Ku80 detection, and 250 ng for histone H3 detection. To visualize specific DPCs, the membrane was immunoblotted with anti-TOP2 (Abcam, ab52934, 1:1000), anti-Ku80 (Cell Signaling, #2753, 1:1000) or anti-histone H3 primary antibodies (Cell Signaling, #9715, 1:2000). After overnight incubation at +4°C with the appropriate primary antibody, visualization of specific signals was performed in the same manner as described in the Western blot analysis. To verify the DNA quantifications, dot blot analysis was performed using a nylon membrane (RPN303B, GE Healthcare). DNA (2 ng) was applied to the nylon membrane, and DNA was detected with α-dsDNA antibody (abcam ab27156, diluted 1:7000, incubated overnight at + 4°C) and the HRP-coupled anti-mouse secondary antibody (A9044, Sigma-Aldrich. 1: 10000) (1 h at room temperature).

### Western blot analysis of yH2AX levels

Zebrafish embryo (2 dpf) lysates collected for Tdp2 activity assay were also analyzed by Western blotting to determine yH2AX levels. In all samples, SDS was added to a final concentration of 0.5%, followed by incubation on ice for 30 min. For Western blot analysis, 5 μg of total protein solutions were boiled for 5 min at 95°C with 5x Laemmli buffer (50 mM Tris-HCl, pH 6.8; 2% SDS; 10% w/v glycerol; 0.05% bromophenol blue; and 5% β-mercaptoethanol). Samples were separated on SDS-PAGE gradient gels (5%–18%) using the Mini-PROTEAN 3 Cell electrophoresis chamber (Biorad). The separated proteins were then transferred to a polyvinylidene difluoride membrane (PVDF, 03010040001, Roche) using the Mini Trans-Blot Cell transfer system (Biorad) via wet transfer at 100 V for 1h and 15 min (0.025% SDS). Blocking was performed using 5% low-fat milk (T145.1, Carl Roth) in TBST (10 mM Tris-HCl (pH 7.5), 15 mM NaCl, 0.02% Tween 20) with gentle rocking for 2 h at room temperature. The membranes were then washed and incubated overnight at 4°C with anti-yH2AX antibody (Abcam, ab81299, 1:2500) in 2.5% BSA TBST buffer or anti-Tubulin antibody (Santa Cruz, sc-134238, 1:7000) which was used as a loading control. The following day, membranes were washed three times for 5 min with TBST buffer and incubated for 1 h with a secondary antibody: goat anti-rabbit IgG-HRP (Sigma-Aldrich, a0545, 1:100,000) for yH2AX and goat anti-mouse IgG-HRP (SigmaAldrich, a9044, 1:100,000) for tubulin, while gently rocking at RT. The membranes were then washed three times for 15 min with TBST buffer and once with TBS (10 mM Tris-HCl (pH 7.5), 15 mM NaCl) buffer. Proteins were detected using ECL blotting substrate (1705061, Biorad) and visualized using the ChemiDoc™ XRS + System (Biorad). Protein size was estimated by use of protein marker (1610374, Biorad).

### Phenotype description

Phenotypes were observed and recorded at 2 dpf. Embryos were dechorionated manually and images were taken using a Samsung 13-megapixel camera with an f/1.9 aperture applied to the ocular of Motic SMZ-171 binocular.

### Statistical analysis

Quantification of silver-stained gels, Dot blots, Western blots, and agarose gels (PCRs) for the evaluation of morpholino-mediated silencing efficiencies was conducted using the ImageJ software (Abràmoff et al., 2004). Graphical representation of the expression data and statistical analysis were conducted using the unpaired two-tailed Student’s t-test with GraphPad Prism 8 software. Statistical significance was considered when *p* < 0.05, indicating differences between two independent conditions. Each experiment was repeated three times, and the results are presented in each column as mean ± standard error (SEM).

## Results

### Comparison of human and zebrafish TDP2

**FIGURE 1:**
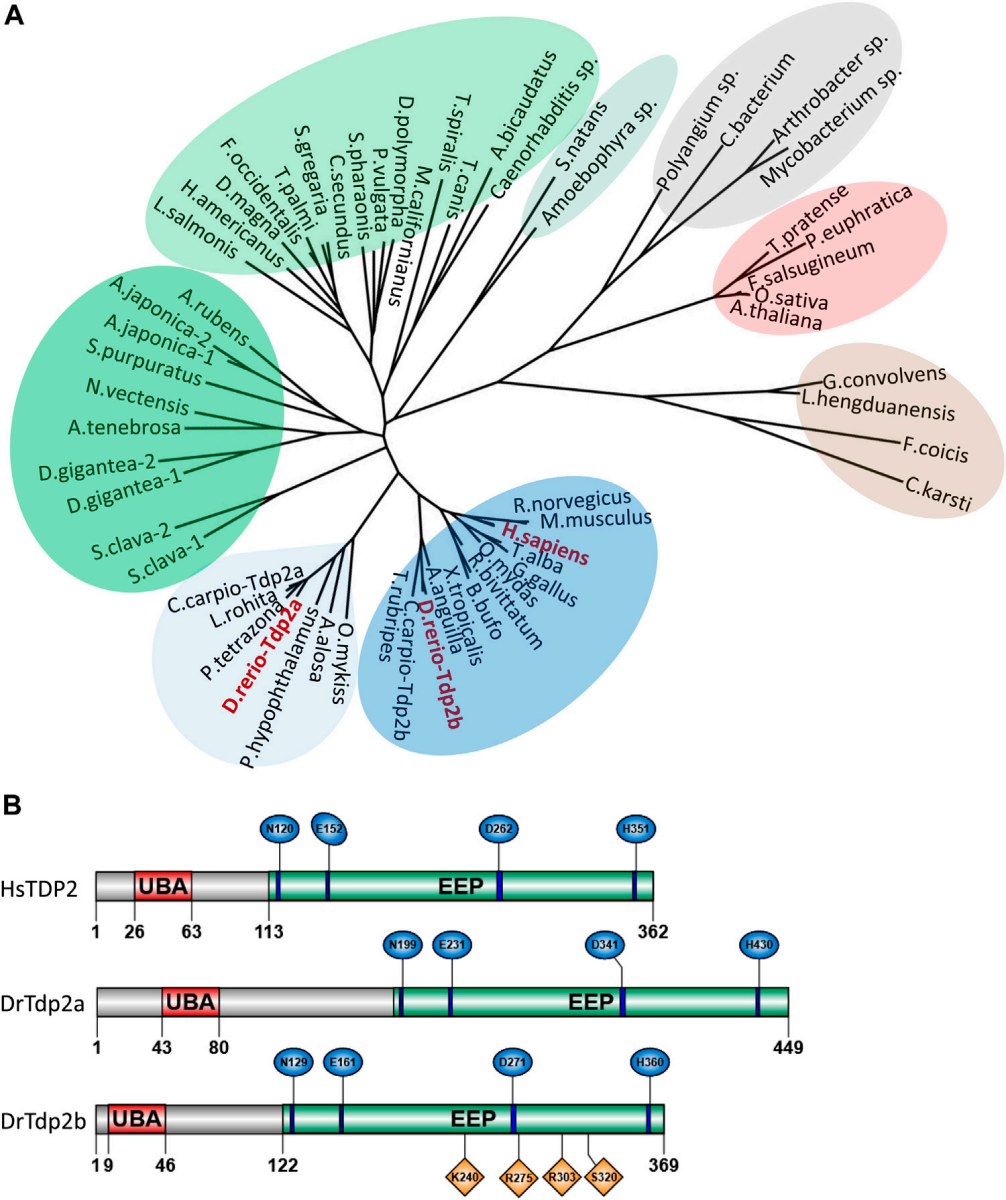
Phylogenetic analysis and domain organization of tyrosyl-DNA phosphodiesterase 2 in humans and zebrafish. **(A)** Phylogenetic tree of tyrosyl-DNA phosphodiesterase 2 (TDP2). Vertebrate orthologs are shown in blue with an additional cluster of Tdp2 co-orthologs in fish is shown in light blue. Two clusters of invertebrate orthologs are shown in green, algae in light green, plant orthologs in red, fungi in brown, and bacterial cluster in grey. Phylogenetic analysis was performed using the Maximum Likelihood method. **(B)** Domain structures of human and zebrafish tyrosyl-DNA phosphodiesterase 2 (UBA - ubiquitin-associated domain; EEP-exonuclease/endonuclease/phosphodiesterase catalytic domain). Conserved catalytic motifs bearing catalytic residues are shown in blue and DNA binding sites in Tdp2b are shown in orange.

Tyrosyl-DNA phosphodiesterase 2 (TDP2) is a highly conserved protein found in all domains of life, including bacteria, fungi, algae, plants, and animals (Figure 1A and Supplementary Figure S1). Yeasts lack the TDP2 protein (Figure 1A), as previously reported (Ledesma et al., 2009). Since TDP2 is an evolutionarily ancient protein, it has likely been lost in yeast lineages during evolution. We observed occasional TDP2 duplications over the course of evolution, specifically in some cyprinid species, including zebrafish and European carp (*Cyprinus carpio*) and within the invertebrate group which is phylogenetically closer to vertebrates (Figure 1A, in dark green), specifically in tunicates (*Styela clava*), echinoderms (*Anneissia japonica*) and cnidarians (*Dendronephthya gigantea*). Due to the teleost-specific whole genome duplication (WGD) event around 320 million years ago (Jatllon et al., 2004), zebrafish often have two paralogs corresponding to a single gene in other vertebrate species (Ravi and Venkatesh, 2008), as is the case for *tdp2a* and *tdp2b*, which are paralogs of human *TDP2* (Figure 1A). Phylogenetic analysis showed that Tdp2b is closer to TDP2 in mammals and other vertebrates, whereas the Tdp2a cluster in teleost fish diverged from the main vertebrate cluster (Figure 1A, in light blue).

**FIGURE 2:**
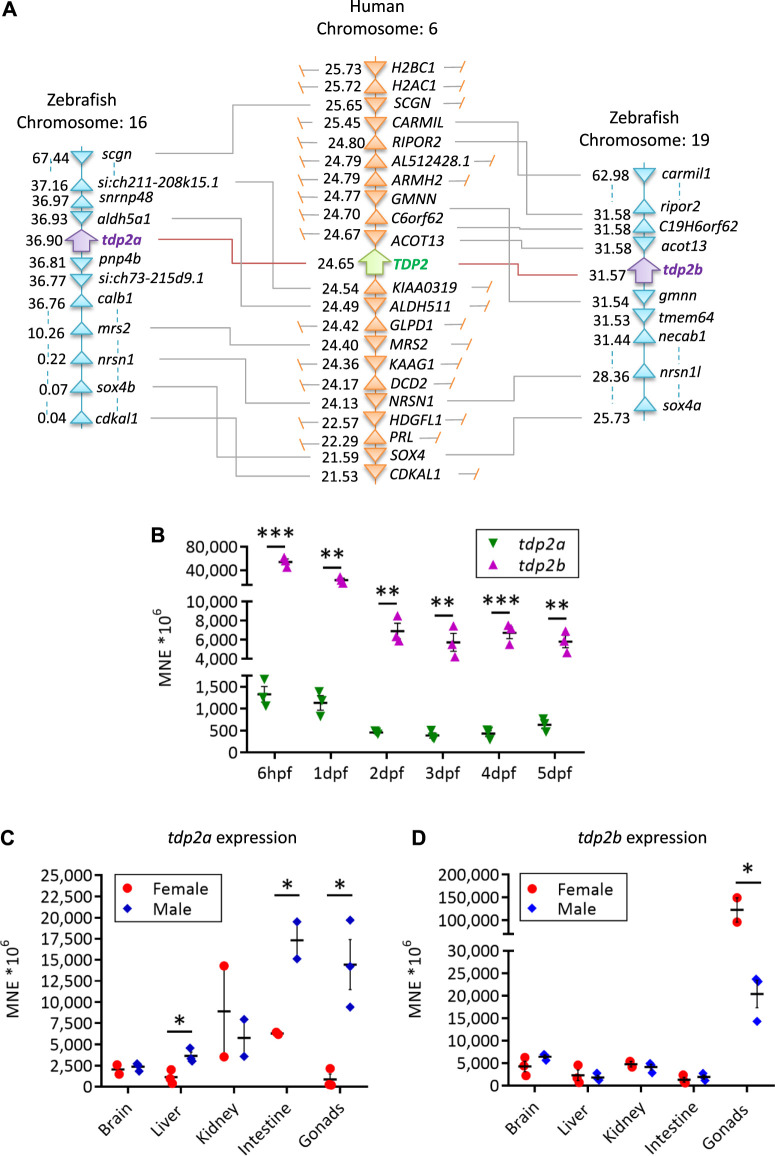
Syntenic analysis of human and zebrafish TDP2 and mRNA expression patterns in embryos and adults. **(A)** Synteny analysis of zebrafish and human *TDP2* genes. The schematic shows the chromosomal positions of the zebrafish and human *TDP2* genes as determined using the Genomics database. The numbers next to the gene names indicate their respective position in megabase pairs (Mbp) on the respective chromosome. **(B)** mRNA expression profiles of *tdp2a* and *tdp2b* during zebrafish embryonic development from 6 h post-fertilization (6 hpf) to 5 days post-fertilization (5 dpf), normalized to the housekeeping gene ATP synthase peripheral stalk (*atp50*). **(C)** Tissue expression pattern of *tdp2a* and **(D)** *tdp2b* in adult zebrafish: brain, liver, kidney, intestine and gonads. Statistically significant differences (*p < 0.05, **p < 0.01, ***p < 0.001) were determined by unpaired *t*-test. Data are presented as MNE (mean normalized expression) ± SEM (*n* = 3), normalized to the housekeeping gene ATP synthase peripheral stalk subunit (*atp50*).

The domain structure is highly conserved between human and zebrafish TDP2 orthologs (Figure 1B) and consists of the N-terminal non-canonical UBA (ubiquitin-associated) domain and the C-terminal catalytic exonuclease/endonuclease/phosphodiesterase (EEP) domain with four conserved catalytic motifs (Figure 1B, in blue) and residues N120, E152, D262 and H351, which form the magnesium coordination site (Schellenberg et al., 2012; Shi et al., 2012). Zebrafish Tdp2b is more similar to human TDP2 than Tdp2a, which has a longer N-terminal part (Figure 1B) that is mostly unstructured (Schellenberg et al., 2012; Shi et al., 2012), and is overall a longer protein compared to Tdp2b and human TDP2 (Figure 1B). The human *TDP2* gene is located on chromosome 6, whereas in zebrafish *tdp2a* is located on chromosome 16, and *tdp2b* on chromosome 19 (Figure 2A). The syntenic analysis showed that the gene environment is conserved between human and zebrafish *Tdp2*. A comparison of the genes surrounding *TDP2* in humans and zebrafish showed that a gene cluster consisting of *ACOT13*, *C6orf62*, and *GMNN* is located upstream of human *TDP2*, which is also found in the vicinity of the zebrafish *tdp2b* gene (Figure 2A). In addition, the *RIPOR2* and *CARMIL* genes are located upstream of zebrafish *tdp2b* (Figure 2A). On the other hand, downstream of human *TDP2* is a gene cluster containing *KIAA0319* and *ALDH511*, which is found upstream of zebrafish *tdp2a* (Figure 2A). Similarly, downstream of human *TDP2*, we found *MRS2* and *NRSN1*, which are located further downstream of zebrafish *tdp2a* (Figure 2A). Interestingly, this small chromosomal region surrounding *TDP2* shows gene duplication similar to the *tdp2* gene. For example, the downstream gene *NRSN1* has two orthologs: *nrsn1* downstream of *tdp2a* and *nrsn1l* downstream of *tdp2b* (Figure 2A). The same is true for the downstream gene *SOX4*, which has two orthologs: *sox4b* downstream of *tdp2a* and *sox4a* downstream of *tdp2b* (Figure 2A). In summary, the gene environment of Tdp2 is partly conserved in humans and zebrafish.

### *Tdp2b* is expressed more strongly than *tdp2a* during embryonic development

In the first 4 hours of vertebrate embryonic development, rapid cell divisions occur without distinct G1 and G2 phases (Siefert et al., 2015). At this stage, maternally deposited mRNAs play a crucial role in early development, including the mRNAs of DNA repair genes (Zhang et al., 2014). Some DNA repair activity is present in zygotes and early-stage embryos, but their ability to recognize and respond to DNA damage is limited (Dey et al., 2023). After 6 h post-fertilization (hpf), maternal transcripts are mostly degraded, and embryonic transcription is fully active (Mathavan et al., 2005). We investigated the expression dynamic of both zebrafish *tdp2* orthologs, *tdp2a* and *tdp2b*, in different embryonic stages ranging from 6 hpf to 5 days post-fertilization (dpf) (Figure 2B). Expression levels were measured using predetermined thresholds (Lončar et al., 2016; Anticevic et al., 2023), where high expression was considered when the normalized expression (MNE) was >60 × 10^6^ (Ct values <22), moderate when MNE is 2 × 10^6^–60 × 10^6^ (Ct = 23–26), and low when MNE is <2 × 10^6^ (Ct > 27). Both *tdp2* orthologs showed high expression, but *tdp2b* exhibited 10 to 40 times higher expression compared to *tdp2a* throughout zebrafish development (Figure 2B). Interestingly, both genes exhibited similar expression patterns, with the highest expression observed at the 6 hpf stage, which gradually decreased and reached a stable expression level at 2 dpf (Figure 2B). Both genes were significantly more expressed in earlier developmental stages (6 hpf and 1 dpf) compared to the later stages (2 – 5 dpf): *tdp2a* three times more and *tdp2b* eight times more (Figure 2B).

### *Tdp2a* and *tdp2b* are both expressed in adult tissues

Using the same gene expression quantification method, we found that *tdp2a* and *tdp2b* are both highly expressed in adult tissue including gonads, brain, kidney and intestine, while their expression is moderate in liver (Figures 2C, D). Notably, both genes showed highest expression in gonads with pronounced gender differences. *Tdp2a* is very highly expressed in testes, aprox. 50-fold more than in ovaries (*p* < 0.1) (Figure 2C). In contrast, *tdp2b* is very highly expressed in ovaries: 5 times higher than in testes (*p* < 0.1) (Figure 2D). Another difference in expression between the two orthologs was observed in the intestinal tissue where *tdp2a* is more highly expressed in both genders (10 times higher than *tdp2b*). In brain, both orthologs exhibited very high expression, followed by high expression in kidney and moderate expression in liver (Figures 2C, D).

### *Tdp2b* gene silencing reduces total Tdp2 activity in zebrafish embryos

**FIGURE 3:**
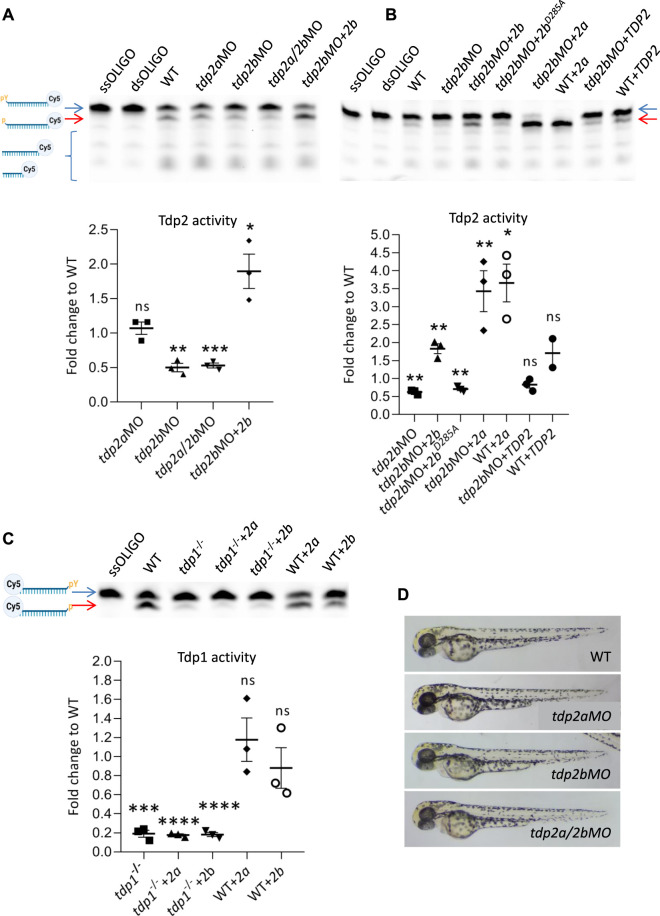
The effects of *tdp2* silencing and overexpression on Tdp1 and Tdp2 enzymatic activity and phenotype in zebrafish embryos. (A) Tdp2 activity in 2 dpf zebrafish embryos after silencing *tdp2a, tdp2b* or *tdp2a/2b*, and overexpression of *tdp2b* in a *tdp2b*-silenced background. Upper panel shows the processing of a Tdp2-specific substrate (5′ (pY)) (blue arrow) after incubation with embryo lysate (10 μg), with ssOLIGO and dsOLIGO as negative controls. The scheme illustrates the reaction products, indicating the Tdp2-mediated removal of tyrosine from the 5′ end (p). Reduced Tdp2 activity is reflected by a lower band intensity of the 5′end (p) product (red arrow). Lower panel shows the quantification of the enzymatic reactions. Tdp2 activity was calculated as the ratio between the band intensity of the lower band (5′(p), 3′ (Cy5), red arrow) and the upper unresolved band (5′ (PY), 3′ (Cy5), blue arrow) for each sample. **(B)** Tdp2 activity in zebrafish embryos after overexpression of catalytically inactive Tdp2b^D285A^, Tdp2a, or human TDP2 in a *tdp2b*-silenced background (upper panel) with the corresponding quantification (lower panel). **(C)** Tdp1 activity in WT and *tdp1* mutant embryos (2 dpf) with or without overexpression of Tdp2a or Tdp2b. The scheme shows the Tdp1 substrate oligonucleotide with a tyrosine (pY) at the 3′end and Cy5 at the 5′end, as well as the reaction product following Tdp1-mediated removal of the tyrosine (p) (upper panel) and corresponding quantification (lower panel). Activities are calculated as the ratio between the band intensity of the lower band (3′(p), 5′ (Cy5), red arrow) and the upper unresolved band (3′ (pY), 5′ (Cy5), blue arrow) for each sample from three biological replicates. Schemes of Tdp1 and Tdp2 substrates were created using BioRender.com. The activity data in A, B and C represent the mean fold change from activity observed in WT embryos ±SEM (*n* = 3). Statistical significance was determined using an unpaired Student’s t-test (* (*p* < 0.05), ** (*p* < 0.01), *** (*p* < 0.001)). **(D)** Representative pictures of live zebrafish embryos at 2 dpf. Morphological changes were not observed after *tdp2a* silencing using morpholino oligonucleotides (*tdp2a*MO), *tdp2b* silencing (*tdp2b*MO), and simultaneous silencing of *tdp2a* and *tdp2b* (*tdp2a/2b*MO).

To investigate the function of *tdp2a* and *tdp2b* in zebrafish embryos, we designed a splice-blocking morpholino to specifically silence *tdp2a* gene expression and a translation-blocking morpholino to specifically inactivate *tdp2b*. We determined the silencing efficiency of the *tdp2a* splice-blocking morpholino by performing PCRs on cDNA derived from 2 dpf embryos (Supplementary Figures S2A, B). No PCR amplification was observed in the *tdp2a* morphant samples, indicating 100% silencing efficiency of the *tdp2a* morpholino (Supplementary Figure S2B). The silencing of *tdp2b* was confirmed by measuring the reduction in Tdp2b enzymatic activity in five independent experiments (biological replicates) (Figures 3A, B; Supplementary Figures S3A, B, E).

To measure the enzymatic activity of Tdp2a and Tdp2b in zebrafish embryos, we performed a TDP2 activity assay (Zeng et al., 2012; Zagnoli-Vieira et al., 2018). In this assay, a Cy5-labelled oligonucleotide containing a 5′phosphotyrosyl moiety (5′-PY) was incubated with whole embryo lysates (Zaksauskaite et al., 2021) of WT, *tdp2a* or *tdp2b* morphants (Figures 3A, B and Supplementary Figures S3A, B, E). To generate a specific substrate for Tdp2, the labelled oligonucleotide was annealed with a complementary primer, resulting in a double-stranded substrate oligomer with a 5′overhang containing the tyrosine residue. In the presence of active Tdp2, the tyrosine residue is removed from the 5′end of the oligomer, resulting in a cleavage product seen as an additional band (p-oligo-Cy5, red arrow) (Figure 3A). In WT embryos, in agreement with previous findings (Zaksauskaite et al., 2021), we observed successful processing of the phosphotyrosyl moiety into a phosphate group (red arrow, Figure 3A). Interestingly, we also detected bands that were lower than the band with the phosphate group (oligo-Cy5), suggesting additional cleavage events (Figure 3A). When comparing Tdp2 activity in embryos, we did not detect significant changes in the specific band intensity between WT embryos and *tdp2a*-silenced embryos (Figure 3A). In contrast, incubation of the substrate with embryos in which *tdp2b* was silenced resulted in a notable 50% reduction in Tdp2 activity (Figures 3A, B and Supplementary Figures S3A, B, E). Similarly, simultaneous silencing of *tdp2a* and *tdp2b* in zebrafish embryos resulted in a reduction of Tdp2 activity compared to silencing of *tdp2b* alone (Figure 3A).

To test the specificity of silencing, we co-injected mRNA encoding full-length Tdp2b with *tdp2b*MO and observed a complete rescue of Tdp2 activity (Figures 3A, B and Supplementary Figures S3A, B, E). Overexpression of *tdp2b* not only restored the loss of Tdp2 activity but also increased the substrate processing by nearly two-fold, resulting in a more intense signal of the oligonucleotide lacking phosphotyrosyl (p-oligo-Cy5) (Figures 3A, B and Supplementary Figures S3A, B, E). As expected, overexpression of catalytically inactive Tdp2b with a mutation in the active site (D285A) could not rescue the activity and showed same activity levels as *tdp2b* morphants (Figure 3B, Supplementary Figures S3A, B). Overexpression of *tdp2b* in both WT and *tdp2b*-silenced embryos resulted in a similar level of Tdp2 activity (Supplementary Figures S3E). Altogether, these results suggest that the Tdp2b ortholog accounts for the overall Tdp2 activity in zebrafish embryos.

*Tdp2a, tdp2b* and *tdp2a/b* morphants showed no visible phenotype at 2 dpf (Figure 3D and Supplementary Figure S4). We conclude that transient impairment of Tdp2 function does not cause abnormalities during early embryonic development.

The Tdp2a ortholog is enzymatically active, but does not contribute to the overall Tdp2 activity in 2-days old zebrafish embryos (Figure 3A). The total Tdp2 activity measured after *tdp2a* silencing was the same as in WT embryos, and is a consequence of active Tdp2b in these samples (Figure 3A). However, overexpression of Tdp2a significantly increased processing of Tdp2 substrate in both WT and *tdp2b*-silenced embryos (Figure 3B, Supplementary Figures S3A, B), showing that the Tdp2a ortholog is enzymatically active. Overexpression of human TDP2 did not result in a significant increase in the substrate processing (Figure 3B, Supplementary Figures S3A, B) which could be a consequence of differences in codon usage between human and zebrafish (Nakamura et al., 2000; Plotkin and Kudla, 2011; Bazzini et al., 2016; Wu et al., 2019). Indeed, analysis of codon usage frequencies for the human TDP2 CDS showed that out of 57 codons in the HsTDP2 CDS, six are significantly underrepresented, and 3 are somewhat underrepresented in zebrafish when compared to human codon frequency (Nakamura et al., 2000).

In addition, we investigated whether Tdp2 zebrafish orthologs can cleave a Tdp1 substrate in zebrafish embryos, given that partial redundancy of Tdp1 and Tdp2 was previously observed (Shimizu et al., 2023). To this end, we used the Tdp1-deficient zebrafish line which we previously created and characterized (Anticevic et al., 2023) and Tdp1 activity assay (Yang et al., 1996). Overexpression of Tdp2a or Tdp2b in Tdp1-deficient and in WT embryos did not result in increased Tdp1 substrate cleavage (Figure 3C, Supplementary Figures S3C, D). All injected mRNAs were stable and highly expressed, as verified by qPCR (Supplementary Figure S2C). In addition, overexpression of different constructs did not affect the morphology and development of embryos (Supplementary Figure S4).

### Tdp2b deficiency causes significant accumulation of DNA-protein crosslinks

**FIGURE 4:**
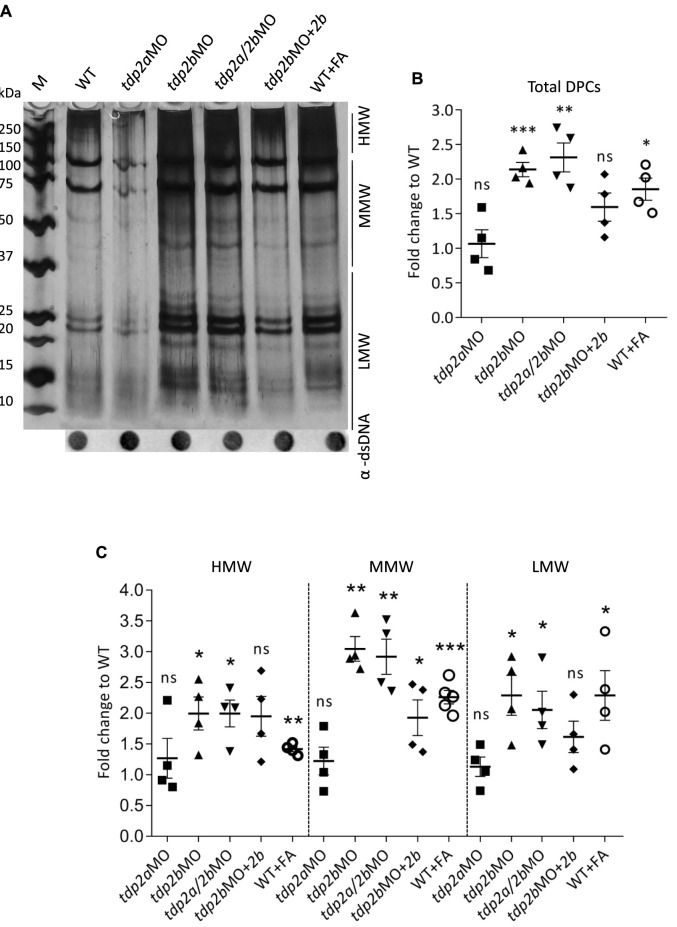
DPC analysis after *tdp2* gene silencing and Tdp2b overexpression in zebrafish embryos. **(A)** DPC analysis for the following conditions: *tdp2a*-silenced embryos (*tdp2a*MO); *tdp2b*-silenced (*tdp2b*MO); *tdp2a*- and *tdp2b*-silenced (*tdp2a/2b*MO) and Tdp2b overexpression in *tdp2b*-silenced embryos (*tdp2b*MO + *tdp2b*). DPCs were isolated from 2 dpf embryos using the RADAR assay (20-30 embryos per condition, *n* = 4), resolved on an SDS acrylamide gel, and visualized by silver staining. Dot-blots show DNA loading controls. WT embryos treated with formaldehyde (10 mM, 30 min) were used as a positive control for DPC induction. **(B)** Quantification of total DPCs from **(A)**. **(C)** Quantification of DPCs from **(A)** according to their molecular weight: High Molecular Weight (HMW) (>150 kDa), Medium Molecular Weight (MMW) (40 kDa–150 kDa), and Low Molecular Weight (LMW) DPCs (protein size <40 kDa). The data represent the mean fold change from WT ± SEM (*n* = 4). Statistical significance was determined using an unpaired Student’s t-test (* (*p* < 0.05), ** (*p* < 0.01) and *** (*p* < 0.001)).

Total DPCs were isolated from wild-type (WT) and *tdp2*-silenced embryos at 2 days dpf post-fertilization and conclusions were derived from four biological replicates. To compare DPC levels in tdp2-deficient embryos with DPC levels induced by a strong, model inducer, formaldehyde (FA), WT embryos were treated with 10 mM formaldehyde (30 min, 28°C) in each experiment. We previously optimized FA exposure conditions to induce DPCs without effects on embryonic phenotypes (Anticevic et al., 2023). Silencing of *tdp2b* led to a 2.1-fold increase in DPC accumulation compared to WT embryos, whereas silencing of *tdp2a* did not significantly affect cellular DPC levels (Figure 4 and Supplementary Figures S5A, B). Simultaneous silencing of *tdp2a* and *tdp2b* resulted in the same DPC increase as silencing of *tdp2b* alone (2.2-fold increase) (Figures 4A, B, Supplementary Figures S5A, B), while FA caused a 1.8-fold increase (Figures 4A, B, Supplementary Figures S5A, C).

In a more detailed analysis of the accumulated DPCs, we categorized the DPCs into three subgroups based on their molecular weight as previously described (Anticevic et al., 2023): High Molecular Weight (HMW >151 kDa), Medium Molecular Weight (MMW, 41 kDa–150 kDa), and Low Molecular Weight (10–40 kDa) (Figure 4C). We are aware that this classification is not ideal, but it helps forming new hypotheses about the repair of DPCs by TDP2. For example, this classification showed that TDP2 deficiency causes the largest increase in protein crosslinks of medium molecular weight (MMW) (Figure 4C), suggesting that TDP2 might be involved in the repair of DPCs in that size range.

Silencing of *tdp2a* did not have significant effects on DPCs in any size range compared to WT embryos (Figure 4C, Supplementary Figures S5A, B). However, significant differences in the MMW and LMW DPC levels were observed in *tdp2b*-silenced embryos, which showed a 3-fold (*p* < 0.01) and 2.3-fold (*p* < 0.05) increase in MMW and LMW DPCs, respectively, compared to WT embryos (Figure 4C).

Silencing both zebrafish *tdp2* orthologs resulted in DPC accumulation comparable to silencing of *tdp2b* alone, evidenced by a 3-fold (*p* < 0.01) increase in the MMW and a 2.3-fold (*p* < 0.05) increase in the LMW DPC range, respectively. Simultaneous silencing of *tdp2a* and *tdp2b*, as well as silencing of *tdp2b* alone, showed similar effects on MMW and LMW DPCs as formaldehyde (FA) treatment, resulting in a 2.2-fold (*p* < 0.001) and 2.3-fold (*p* < 0.01) increase, respectively, compared to WT levels. Silencing of *tdp2b* also led to an accumulation of HMW DPCs, showing a 2-fold change (*p* < 0.05), reflecting the same increase observed in *tdp2a*/*b* double morphants (2-fold, *p* < 0.05). This increase was again comparable to that observed when DPCs were induced by FA, resulting in a 1.4-fold increase (*p* < 0.001) (Figures 4A, C; Supplementary Figures S5A, B).

To confirm that the increase in DPC levels was specifically due to loss of Tdp2b, we measured DPC accumulation following Tdp2b overexpression in *tdp2b*-silenced embryos. Indeed, co-injection of Tdp2b mRNA with the *tdp2b* morpholino was able to partially reduce total DPC levels (from 2.1-fold to 1.5-fold); MMW and LMW DPC levels were reduced from 3-fold to 2-fold (*p* < 0.05) and from 2.3-fold to 1.8-fold, respectively, while HMW DPC levels were unaffected (Figure 4 and Supplementary Figures S5A–C).

**FIGURE 5:**
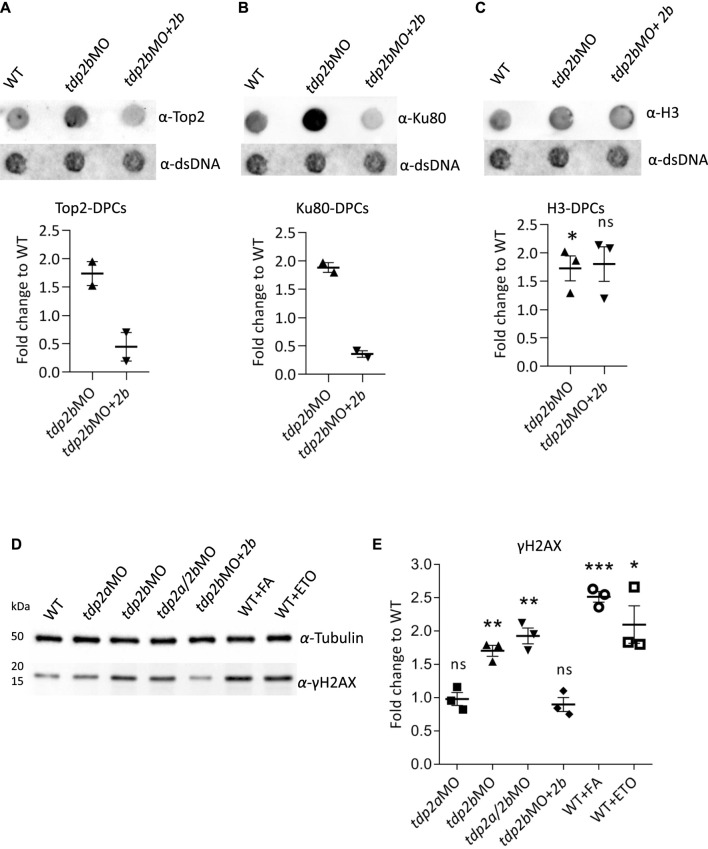
Silencing of *tdp2b* causes accumulation of Top2-DPCs, Ku80-DPCs and DSBs. **(A)** Dot blots and corresponding quantifications normalized to WT embryos showing **(A)** Top2-DPCs (*n* = 2), **(B)** Ku80-DPCs (*n* = 2), and **(C)** histone H3-DPCs (*n* = 3) with corresponding DNA loading controls, after *tdp2b* silencing (*tdp2b*MO) or Tdp2b overexpression in *tdp2b*-silenced embryos (*tdp2b*MO+*2b*). **(D)** Western blot analysis of γH2AX levels in WT embryos (WT), *tdp2a*-silenced (*tdp2a*MO), *tdp2b*-silenced (*tdp2b*MO); *tdp2a*- and *tdp2b*-silenced (*tdp2a/2b*MO) and in embryos where Tdp2b was overexpressed in *tdp2b*MO (*tdp2b*MO+*2b*). Tubulin was used as a loading control. WT embryos treated with formaldehyde (10 mM, 30 min) or etoposide (50 μM, 1 h) were used as a positive control for DSB induction. **(E)** Quantification of **(D)**. Data show the mean fold change compared to WT ± SEM (*n* = 3). Statistical significance was determined using an unpaired Student’s t-test (* (*p* < 0.05), ** (*p* < 0.01) and *** (*p* < 0.001)).

We further analysed the DPC isolates in order to identify which crosslinked proteins were accumulated in zebrafish embryos as a consequence of Tdp2b silencing. The levels of a known substrate of Tdp2b, Top2, were increased by 1.7-fold after Tdp2b silencing, and the overexpression of Tdp2b in the *tdp2b*-silenced embryos resulted in the complete rescue of the observed Top2-DPC increase (Figure 5A and Supplementary Figure S5D). Other DPC substrates of Tdp2 are currently unknown. We decided to test if two other DPCs which are known to be one of the most abundant in the cells under physiological condition (i.e., endogenous DPCs), Ku80 and histone H3 (Kiianitsa and Maizels, 2020) are affected by Tdp2b silencing. We observed 1.9-fold increase in Ku80-DPCs as a consequence of *tdp2b* silencing and Tdp2b overexpression rescued the Ku80-DPC levels (Figure 5B and Supplementary Figure S5E). In contrast, histone H3-DPCs accumulated in *tdp2b*-silenced embryos, but overexpression of Tdp2b did not reduce the observed accumulation (Figure 5C and Supplementary Figure S5F).

### Tdp2 deficiency leads to DSB accumulation *in vivo*

We wanted to investigate whether TDP2 deficiency leads to an increase in double strand breaks (DSBs) as it has previously been shown in cell culture that the accumulation of DPCs leads to an increase in DSBs (Vaz et al., 2016). Although it remains unknown whether silencing of *TDP2* in human cells under physiological conditions leads to DSB accumulation, several studies showed a significant increase in DSBs after treatment with etoposide in cells lacking functional TDP2 (Gómez-Herreros et al., 2013; Zagnoli-Vieira et al., 2018).

Phosphorylation of histone H2AX at serine 139 (γH2AX) is an early marker for DSBs and occurs upon recognition of DSBs by the DNA damage-dependent kinases ATM, ATR, and DNA-PK (Paull et al., 2000; Revet et al., 2011). To investigate whether *tdp2* silencing leads to DSB formation at the organismal level, we quantified γH2AX levels after *tdp2a* and *tdp2b* silencing, while WT embryos exposed to the model DSB inducers formaldehyde (FA) (10 mM, 30 min, 28°C) and etoposide (ETO) (50 μM, 1 h, 28°C) were used as positive controls and reference points (Muslimović et al., 2009; Kumari et al., 2012). Silencing of *tdp2a* had no effect on DSB levels in 2 dpf embryos, while silencing of *tdp2b* caused a 1.8-fold increase in γH2AX levels, similar to the effect of simultaneous silencing of *tdp2a* and *tdp2b* (1.9-fold increase) (Figures 5D, E). In comparison, FA and ETO treatments of WT embryos caused a 2.5-fold and 2.1-fold increase in DSBs, respectively (Figures 5D, E).

Since overexpression of Tdp2b successfully rescued Tdp2b activity and total DPC accumulation in Tdp2b-deficient embryos (Figures 3A, B; Figure 4), we investigated whether it could also rescue the pronounced DSB accumulation induced by *tdp2b* silencing (Figures 5D, E and Supplementary Figure S3F). Indeed, Tdp2b overexpression reduced the elevated DSB levels in *tdp2b*-silenced embryos, back to WT levels (Figures 5D, E and Supplementary Figure S3F). Interestingly, overexpression of Tdp2b in WT embryos significantly further reduced γH2AX levels compared to WT (Supplementary Figure S3F).

## Discussion

Studying DNA repair and DNA-protein crosslink repair (DPCR) pathways in zebrafish complements cell models and offers new perspectives, especially since data from vertebrates are still scarce. TDP2, a key player in this pathway, is essential for the resolution of TOP2 DNA-protein crosslinks. We successfully knocked down both *tdp2* genes in zebrafish embryos using morpholino antisense nucleotides, allowing us to analyze DPC levels and the consequences of DPC accumulation at the organismal level. Since zebrafish *tdp2* genes have not been characterized so far, we first performed a comparative analysis of zebrafish and human TDP2 and investigated their phylogenetic relationship, gene environment, protein domains and mRNA expression patterns during vertebrate embryonic development and in adult tissues (Figures 1, 2 and Supplementary Figure S1; Supplementary Table S1). It is known that the zebrafish genome is characterized by a considerable number of duplicated genes (Howe et al., 2013). This process of duplication and retention of duplicated genes was crucial for the expansion of fish genomes, as a fish-specific whole genome duplication (WGD) event occurred 350 million years ago (Meyer and Van de Peer, 2005). Moreover, this process has played a crucial role in the evolutionary divergence of fish and tetrapods, resulting in a greater diversity of fish genes. Some orthologous genes have evolved to take on specialized functions or specific gene expression patterns, contributing to the diverse genetic profile of the fish genome (Liu et al., 2012). A detailed synteny analysis of human and zebrafish TDP2 genes revealed that the downstream gene cluster of human *TDP2* is partially conserved as a downstream cluster of zebrafish *tdp2b* (Figure 2A). Conversely, the upstream gene cluster of human *TDP2* is partially conserved as the upstream gene environment of zebrafish *tdp2a* (Figure 2A). We showed that in addition to *tdp2*, which underwent gene duplication in zebrafish, two other neighboring genes, *nrsn1* and *sox4*, which remained in the upstream gene cluster of human TDP2, were also duplicated and are present upstream of both orthologs, *tdp2a* and *tdp2b* (Figure 2A), while the other neighboring genes were lost after duplication in zebrafish, as was the case for the vast majority (80%) of genes after the WGD event in teleosts (Glasauer and Neuhauss, 2014). This observation provides valuable insights into the ancestral genomic changes in zebrafish and is yet another example of gene duplication event that have shaped their present-day genomic organization.

Of the two zebrafish *tdp2* orthologues, Tdp2b is evolutionarily closer to human TDP2 (Figure 1A and Supplementary Figure S1). Although both zebrafish orthologues have a conserved catalytic domain (Figure 1B), Tdp2b is more similar to human TDP2 when comparing the N-terminal region (Figure 1B). The expression patterns of *tdp2a* and *b* during the vertebrate development indicate that *tdp2b* is more highly expressed than *tdp2a* and very highly expressed starting from 6 hpf, when embryonic transcription begins and maternal transcripts are mostly degraded (Mathavan et al., 2005; Laue et al., 2019). The very high expression of *tdp2b* continues later on in all larval stages (Figure 2B). Such high expression levels of *tdp2b* highlight the importance of *tdp2* in cellular processes, especially during intense replication and transcription rates in a developing embryo. The particularly high expression of *tdp2b* at 6 hpf and 1 dpf compared to later stages indicates a higher requirement for Tdp2-mediated DNA repair at this phase of development. It is worth noting that while *tdp2b* is much more highly expressed than *tdp2a*, *tdp2a* is also present at moderate to high expression levels throughout the development, suggesting that it plays a role in this time frame (Figure 2B). Recent data from the Farrell Lab indicates that *tdp2a* is specifically and strongly expressed in primordial germ cells (PGCs) at 1 dpf (Sur et al., 2023), raising the intriguing possibility of a highly specific role of Tdp2a in those cells.

In adult zebrafish, high expression of both orthologs in gonads, brain, intestine and kidney and moderate expression in liver suggest roles in DNA repair across different tissues. Especially high expression in testes and ovaries (Figures 2C, D) indicates the protective role of *tdp2a* and *tdp2b* in preserving genome integrity during gametogenesis. Curiously, *tdp2a* appears to be more important in the testis, while *tdp2b* is predominantly expressed in the ovaries. This is in line with previously reported data from microarray analyses which showed high *tdp2b* expression in female gonads (Small et al., 2009). It is not uncommon that duplicated genes acquire distinct gene expression patterns or tissue-specific functions during evolution (Rice, 1984; Gnad and Parsch, 2006; Whitehead and Crawford, 2006). Very high expression of both, *tdp2a* and *tdp2b*, in the brain (Figures 2C, D) is consistent with the role of TDP2 in neuronal tissue where it was found to protect transcription against endogenous abortive TOP2 activity including the transcription of numerous genes essential for neurological development and function. This protective function has been observed in cultured human cells derived from TDP2-deficient patients and in post-mitotic mouse neurons following abortive TOP2 activity (Gómez-Herreros et al., 2014). Furthermore, the high expression of *tdp2a* in intestinal tissue (Figure 2C) suggests a protective role of TDP2 in intestine, especially considering that mice lacking TDP2 display intestinal damage and significant weight loss upon etoposide treatment (Gómez-Herreros et al., 2013).

To compare the tissue expression patterns between zebrafish and human *TDP2*, we used the data for mRNA expression of human *TDP2* from human protein atlas (Karlsson et al., 2023, https://www.proteinatlas.org/). *TDP2* exhibits highest expression in the intestine (normalized transcripts per million), followed by the kidney and testis. Sequentially, the liver, brain, and ovaries follow, exhibiting the lowest expressions among the examined tissues. This pattern resembles zebrafish *tdp2a* with the exception of very high expression of *tdp2a* in testes (Figure 2C). To some extent it also resembles the tissue expression pattern of *tdp2b* with the exception of comparatively lower expression of *tdp2b* in intestine and comparatively higher in ovaries (Figure 2D). It is worth noting that human expression data in protein Atlas is heavily biased toward analysis of older individuals above 60 years of age (Uhlén et al., 2015) while zebrafish analysis was done on 1 year-old adults which approximately corresponds to middle age humans (35–45 years old).

In order to analyze the function of *tdp2a* and *tdp2b* in DPCR in zebrafish, we used morpholinos to transiently and efficiently knockdown their expression. Simultaneous silencing of *tdp2a* and *tdp2b* did not cause gross morphological changes in zebrafish embryos (Figure 3D and Supplementary Figure S4). This stands in contrast to a study published in 2007, in which the function of *tdp2b* (but not *tdp2a*) was analyzed using morpholinos and in which it was found that the silenced embryos exhibited pericardial edemas and abnormalities in blood circulation in the trunk and tail region (Esguerra et al., 2007). However, after *in silico* analysis of the morpholino oligonucleotide sequences used in this study, we identified design flaws based on information provided by the manufacturer of the morpholino oligonucleotide, Genetools LLC. Specifically, morpholino oligonucleotide 1 (Esguerra et al., 2007) was designed to bind to exon1 and exon2 which cannot block splicing instead of targeting the exon-intron boundary which can block splicing, while morpholino oligonucleotide 2 (Esguerra et al., 2007) was positioned more than 80 bp downstream of the start codon which is too far for efficient ATG silencing (Moulton, 2007). Due to these design flaws, it is highly unlikely that these morpholino probes can effectively silence *tdp2b*. In fact, the efficiency of silencing has not been confirmed, nor has the specificity of the morpholinos been tested with overexpression of *tdp2b* mRNA. Therefore, we conclude that the phenotypes observed in a previous study by Esguerra et al. (2007) are not due to *tdp2b* deficiency and are likely non-specific, and that Tdp2 deficiency does not cause changes in embryonic phenotypes (Figure 3D and Supplementary Figure S4). In our study, silencing efficiency was confirmed to be very high (Supplementary Figure S2B), and all measured endpoints, including Tdp2b activity, DPC accumulation and DSB accumulation caused by Tdp2b loss of function were restored after overexpression of Tdp2b (Figures 3–5, Supplementary Figures S3, S5). Specificity was further confirmed by overexpression of the catalytically inactive Tdp2b variant (D285A) (Schellenberg et al., 2012), which did not rescue Tdp2 activity (Figure 3B, Supplementary Figures S3A, B).

In the activity assay, we evaluated the ability of *tdp2a* and *tdp2b* to remove tyrosine moiety from the 5′end of DNA, which is used as a proxy for tracking the resolution of the trapped TOP2 peptide remnant (Ledesma et al., 2009; Zeng et al., 2011). *Tdp2b* silencing resulted in a significant 50% decrease in Tdp2 activity, underscoring its crucial role in resolving TOP2-DPCs in zebrafish embryos (Figures 3A, B, Supplementary Figures S3A, B). In contrast, efficient silencing of *tdp2a* did not influence total enzymatic activity (Figures 3A, B), which shows that the active Tdp2b in these samples, accounts for all measured enzymatic activity. This conclusion was also supported by the fact that reduction in activity in *tdp2b* morphants was the same as in *tdp2a/2b* double morphants (Figures 3A, B). The loss of Tdp2 function in zebrafish embryos was successfully restored by overexpressing Tdp2b, but not catalytically inactive Tdp2b (D285A) (Figures 3A, B, Supplementary Figures S3A, B). Although Tdp2a is enzymatically active when overexpressed (Figure 3B, Supplementary Figures S3A, B), under physiological conditions it does not contribute to the total Tdp2 activity in 2-days old zebrafish embryos. However, considering that Tdp2a is enzymatically active and that is expressed in distinct tissues of adult fish (Figure 2C), it most probably also has a function in Top2-DPC repair, and possibly in the repair of other DPCs which remains to be answered in future studies. Overall, these findings provide strong evidence that Tdp2b is a primary 5′end DNA processing enzyme during vertebrate development.

Recently, it was shown *in vitro* and in immortalized cell lines that TDP2 can repair TOP1-DPCs in the absence of TDP1 (Tsuda et al., 2020; Shimizu et al., 2023). However, the repair kinetics of TOP1-DPCs in TDP1 deficient cells was slower than in WT cells, suggesting that TDP2 is much less efficient than TDP1 in eliminating TOP1-DPC remnant in cultured cells. On the other hand, human syndromes give a somewhat different indication. Considering that TDP2 is presumably functional in SCAN1 patients and in TDP1-deficient mice, and that TDP1 is presumably functional in SCAR23 patients and TDP2-deficient mice, while they still develop neurological deficits (Takashima et al., 2002; Hirano et al., 2007; Errichiello et al., 2020), it seems that *in vivo*, these two proteins cannot fully compensate for each other. In this study, we added another piece to the puzzle, as our data shows that overexpression of Tdp2 orthologs does not result in the cleavage of a Tdp1 substrate in zebrafish embryos (Figure 3C, Supplementary Figures S3C, D).

The most surprising result of *tdp2* silencing was the significant increase in total DNA-protein crosslinks (DPCs) ranging in size from 10 to 250 kDa that is similar to DPC levels induced by FA (Figure 4, Supplementary Figures S5A, C). While it is expected for major DPC processing enzymes like SPRTN protease to induce total DPCs when impaired in cell models and *in vivo* (Vaz et al., 2016; Anticevic et al., 2023; Otten et al., 2023), or when exposed to DPC inducers like formaldehyde (Ruggiano et al., 2021; Anticevic et al., 2023), TDP2 has so far been shown to remove only TOP2-DPCs, which have a size of 176 kDa in zebrafish (Ledesma et al., 2009; Gómez-Herreros et al., 2017; Schellenberg et al., 2017; Lee et al., 2018). Overexpression of recombinant Tdp2b protein in *tdp2b*-silenced embryos rescued DPC levels (Figure 4B), confirming that this accumulation, especially in the medium molecular weight range (from 40 kDa to 150 kDa), is specifically due to the loss of *tdp2b* function (Figure 4 and Supplementary Figures S5). Our study therefore suggests additional roles of TDP2 in DPC repair *in vivo*.

The Tdp2b-dependent increase in TOP2-DPCs confirmed the specificity of our experimental setup, as TOP2-DPC is a well characterized TDP2 substrate (Riccio et al., 2020). Whether the majority of TDP2-mediated removal of TOP2-DPCs occurs through proteolysis and TDP2-mediated repair of the remaining crosslink (Vaz et al., 2017; Morimoto et al., 2019), or through the action of the sumo ligase ZATT (ZNF451) and TDP2 (Schellenberg et al., 2017), remains to be determined in future studies. The involvement of TDP2 in Ku70/80-DPC removal has not yet been investigated. Prior to our study it was shown that the ATPase p97/Vcp can extract Ku70/80 dimers from chromatin at the site of DSBs (van den Boom et al., 2016) and that TDP2 and Ku70/80 act epistatically in error-free NHEJ after DSB induction by TOP2 poisons (Gómez-Herreros et al., 2013). As the Ku70/80 dimer is one of the most abundant endogenous DPCs, understanding its repair is of interest to the DDR field and beyond. We also found that the repair of histone-H3 DPCs is not dependent on Tdp2b, which was expected since we and others have recently showed that histone H3-DPCs at AP sites are repaired by TDP1 (Wei et al., 2022; Anticevic et al., 2023).

Our study is the first to investigate the effect of *tdp2* silencing on cellular DPCs in an animal model. These results suggest that Tdp2 is not only important for the resolution of TOP2- DPCs, but also plays a role in the resolution of other DPCs. It is also possible that part of the observed effects is indirect, and stem from the effects of impaired Tdp2 function on other unknown cellular processes or from side effects of impaired TOP2-DPC removal. It will be interesting to investigate the effect of permanent TDP2 deficiency in adult tissues, especially in the brain of zebrafish TDP2 mutants.

High cellular DPC loads that cannot be repaired in time eventually lead to the occurrence of DSBs. Measurement of yH2AX accumulation as a marker for DSB formation in *tdp2b* zebrafish morphants revealed a 1.8-fold increase in DSBs compared to WT embryos. The observed increase in DSBs is striking, considering that a similar 2.5- and 2.1-fold increase was observed in embryos following acute exposure to formaldehyde and etoposide, respectively (Figures 5D, E, Supplementary Figure S3F). It is known that TDP2 deficiency in combination with etoposide treatment, a TOP2 poison, leads to DSB accumulation in cell cultures (Kont et al., 2016) and in mice (Gómez-Herreros et al., 2013). *Tdp2* knockout mice showed increased mortality and increased toxicity in lymphoid tissue when treated with etoposide (Gómez-Herreros et al., 2013). Mouse embryonic fibroblasts (MEFs) from these animals also showed an increased number of DSBs and chromosomal breaks after etoposide treatment. We hypothesize that in the absence of TDP2, DSBs caused by TOP2-DPCs rely heavily on error-prone NHEJ and HR repair pathways (Gómez-Herreros et al., 2013; Kawale and Povirk, 2018). Considering that HR is overall a rare event in tissues as sister chromatids are required, the majority of DSBs are repaired via the error prone NHEJ pathway, resulting in the formation of blunt DNA ends that often lead to insertions or deletions at the break site. TDP2 promotes error-free NHEJ by processing 5′-TOP2 overhangs to generate 4-base-long sticky ends suitable for rejoining (Gómez-Herreros et al., 2013). Repair of DSBs by error-free NHEJ, which typically occurs after TOP2 activity, is impaired in the absence of TDP2. During the embryonic development, rapid cell division and intense transcription activity requires fast and precise DSB repair (Liu et al., 2012; Vierstraete et al., 2017). Without TDP2, this leads to an accumulation of unrepaired DSBs. This hypothesis is supported by our observations that overexpression of Tdp2b in *tdp2b*-silenced, and in WT embryos, leads to a decrease in DSB (Figures 5D, E; Supplementary Figures S3F).

In conclusion, our findings shed light on the consequences of Tdp2 deficiency on DNA repair processes at the organismal level and provide a foundation for further research in this field. Overall, these results contribute to a better understanding of TDP2 role in zebrafish development and in DNA repair pathways, offering new insights for the study of human diseases related to TDP2 dysfunction.

## Data Availability

The data presented in the study are deposited and available here: https://data.fulir.irb.hr/islandora/object/irb:425.

## Supplementary material

Supplementary Figure S1 Phylogenetic tree of tyrosyl-DNA phosphodiesterase 2 (TDP2) corresponding to the tree shown in Figure 1, showing branch support Alrt values (Approximate likelihood-ratio test) for the tree nodes on a scale of 0-1, where 1 indicates maximum node confidence.

Supplementary Figure S2
*Tdp2a* and *tdp2b* morpholino-mediated silencing scheme and *tdp2a*, *tdp2b* or *TDP2* overexpression in injected embryos **(A)** Scheme of the zebrafish *tdp2a* and *tdp2b* genes indicating the sequences targeted by the morpholinos (red arrowheads) and the primers used to determine the morpholino efficiency (blue arrows). Exons are shown as blue squares and UTRs as yellow squares. Since some introns are very long, they are abbreviated in brackets. The *tdp2a* morpholino targets the exon3—intron3 boundary, the *tdp2b* morpholino targets the 5’UTR. **(B)** DNA gel electrophoresis from three different experiments showing the resolution of PCR reactions performed with cDNA from 2 dpf WT and *tdp2a* morphant embryos. The 524 bp band is expected for PCRs on WT samples (arrow). The numbers next to the marker bands indicate the size in kilobases (kb). **(C)** qPCR experiment detecting *tdp2a*, *tdp2b* or *TDP2* overexpression in embryos injected with the corresponding mRNAs. Statistically significant differences (to WT) in embryos injected with the respective mRNAs were determined by unpaired t-test (****p* < 0.001, **** *p* < 0.0001). Data are presented as MNE (mean normalized expression) ± SEM (n = 3), normalized to the housekeeping gene ATP synthase peripheral stalk subunit (atp50).

Supplementary Figure S3 Biological replicates corresponding to Figures 3 and 5 showing the effects of *tdp2* silencing and overexpression on Tdp1 and Tdp2 enzymatic activity and γH2AX levels. **(A)** The second and **(B)** the third experiment showing denaturing PAGE gels demonstrating the processing of a Tdp2-specific substrate in zebrafish embryos. **(C)** The second and **(D)** the third experiment showing the Tdp1 enzymatic activity **(E)** Comparison of Tdp2 activity analysis in WT and *tdp2b*-silenced zebrafish embryos with or without overexpression of recombinant Tdp2b (mRNA). **(F)** Western blot analysis of γH2AX levels in zebrafish embryos (2 dpf) showing WT and *tdp2b*-silenced embryos with and without *tdp2b* overexpression (mRNA). 5µg of total protein lysate was separated on a gradient polyacrylamide gel (5–18%), with Tubulin as loading control.

Supplementary Figure S4 Additional pictures of live zebrafish embryos at 2 dpf. **(A)** Additional overview images of WT and *tdp2*-silenced embryos related to Figure 3D. **(B)** Representative pictures of zebrafish embryos at 2 dpf related to the activity assays shown in Figure 3: WT, WT injected with mRNA encoding Tdp2bD285A, Tdp2a or HsTDP2, *tdp2b*MO co-injected with mRNA encoding Tdp2b, Tdp2bD285A, Tdp2a or HsTDP2. Morphological changes were not observed in any condition.

Supplementary Figure S5 DPC analysis of biological replicates used to quantify the total DPC levels shown in Figure 4 and 5. **(A)** Second and **(B)** third experiment showing total DPC analysis after silencing of *tdp2a* and/or *tdp2b*, and rescue of *tdp2b*-silenced embryos after overexpression of *tdp2b*. DPCs were isolated from 2-day-old embryos using the RADAR assay (30 embryos per condition), resolved on an SDS acrylamide gel, and visualized by silver staining (equivalent to 250 ng of total DNA loaded per well). Dot-blots show DNA loading controls for DPC analysis prior to benzonase treatment (2 ng of total DNA per well). **(C)** Second and third biological replicate showing DPC levels in WT embryos after FA treatment (10 mM, 30 min) used as a positive control for DPC induction, with corresponding DNA dot blot, used for quantification of DPC levels after FA exposure in WT embryos shown in Figure 4B and C. **(D)** Second biological replicate showing Top2-DPCs used for quantification in Figure 5A (lower panel) **(E)** Second biological replicate showing Ku80-DPCs used for quantification in Figure 5B (lower panel). **(F)** Second and third experiment showing H3-DPCs used for the quantification of histone H3-DPCs shown in Figure 5C (lower panel).

Supplementary Table S1 Accession numbers and species names of protein sequences used for phylogenetic analysis. Sequences were retrieved from the National Center for Biotechnology Information (NCBI) database using the blastp algorithm, with the human TDP2 protein as the query sequence.
