# Supplementary material for: Tyrosyl-DNA phosphodiesterase 2 (Tdp2) repairs DNA-protein crosslinks and protects against double strand breaks in vivo
Source: Front Cell Dev Biol. 2024 Aug 20;12:1394531. doi: 10.3389/fcell.2024.1394531 (PMC11369425; doi:10.3389/fcell.2024.1394531)
Supplement: Supplementary file 1 [file Presentation1.pptx]

## Slide 1
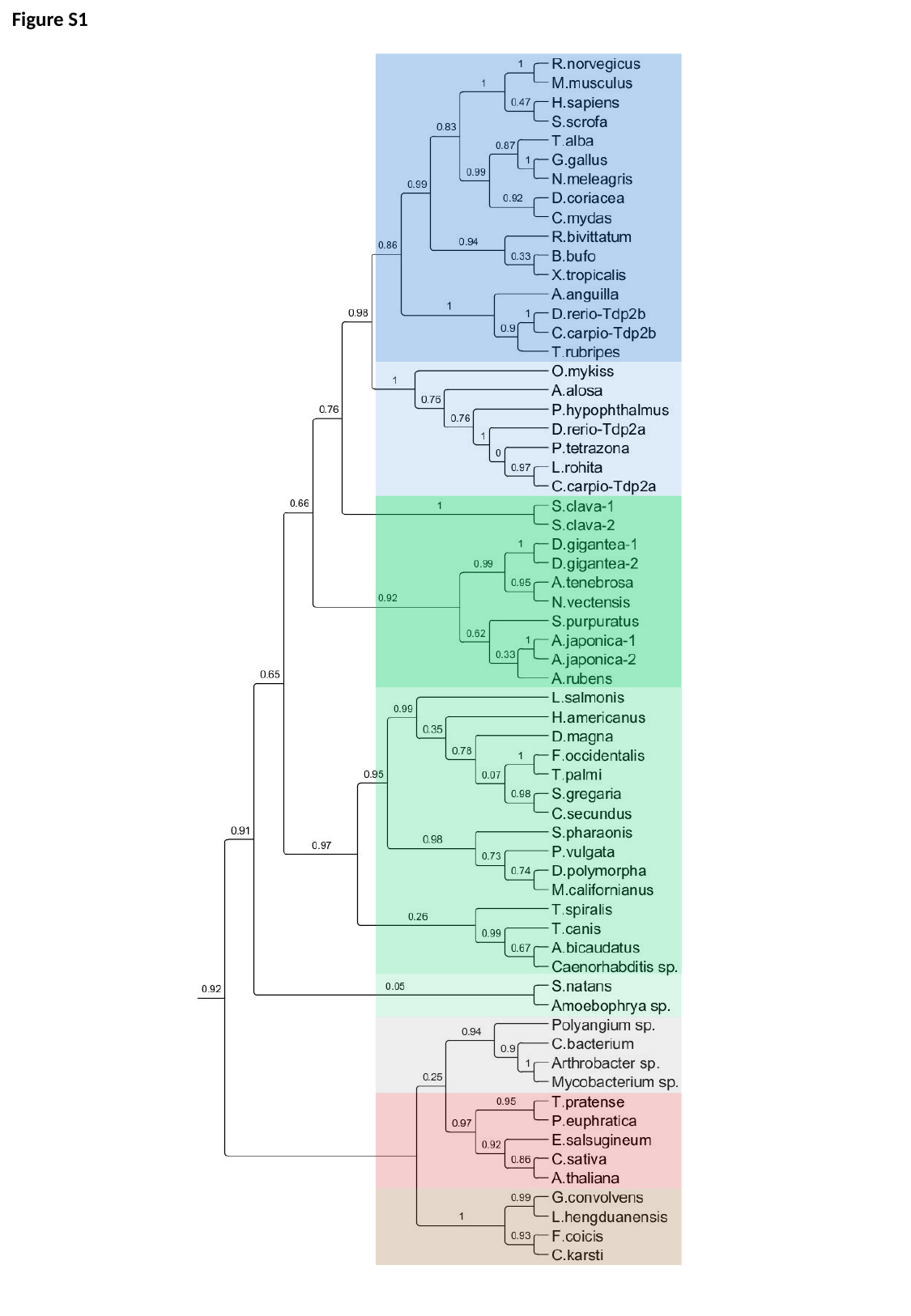

Figure S1

## Slide 2
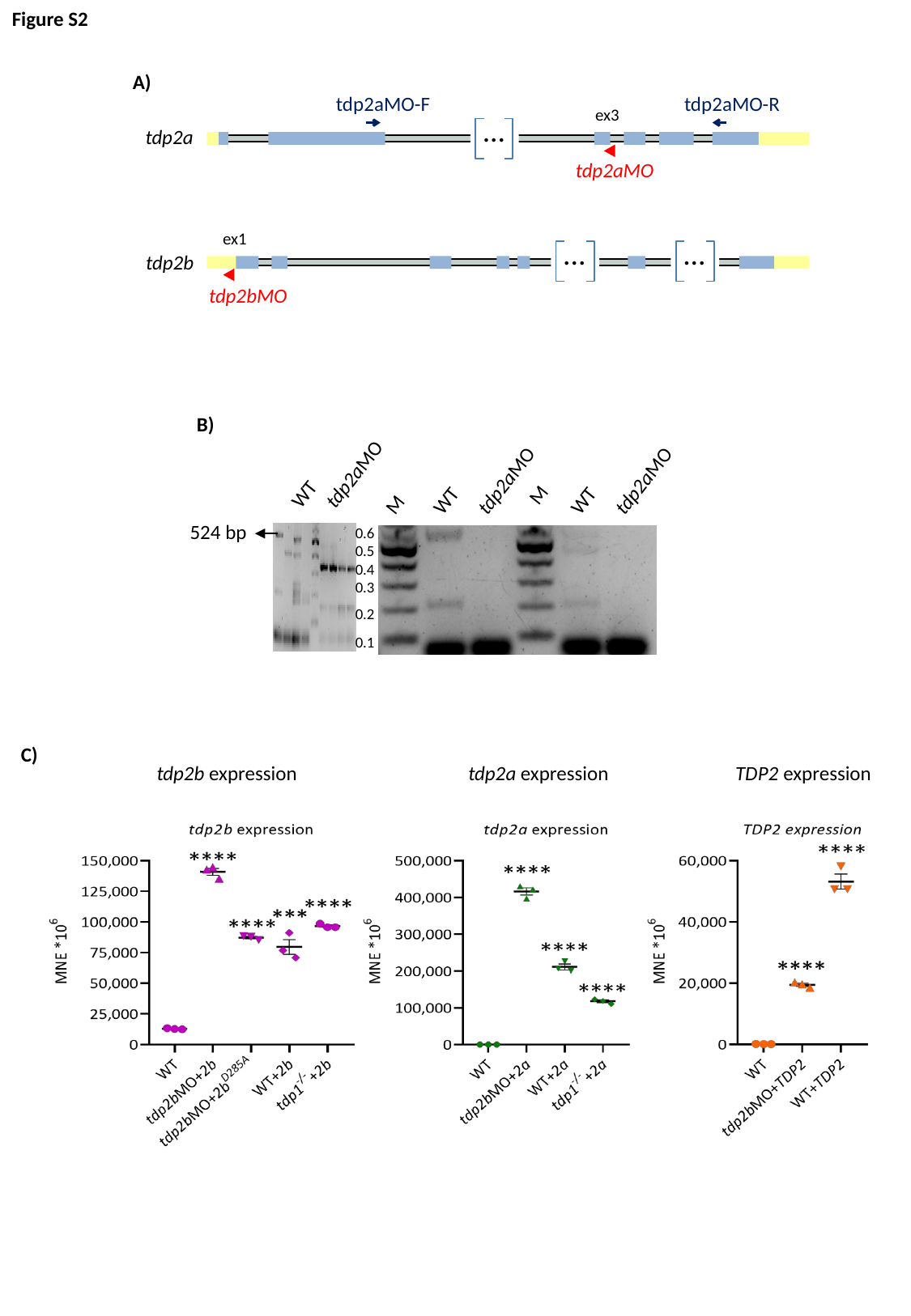

Figure S2
A)
tdp2aMO-F
tdp2aMO-R
ex3
...
tdp2a
tdp2aMO
ex1
...
...
tdp2b
tdp2bMO
WT
tdp2aMO
B)
0.6
0.5
0.40.3
0.2
0.1
524 bp
M
tdp2aMO
tdp2aMO
M
WT
WT
C)
tdp2b expression
tdp2a expression
TDP2 expression

## Slide 3
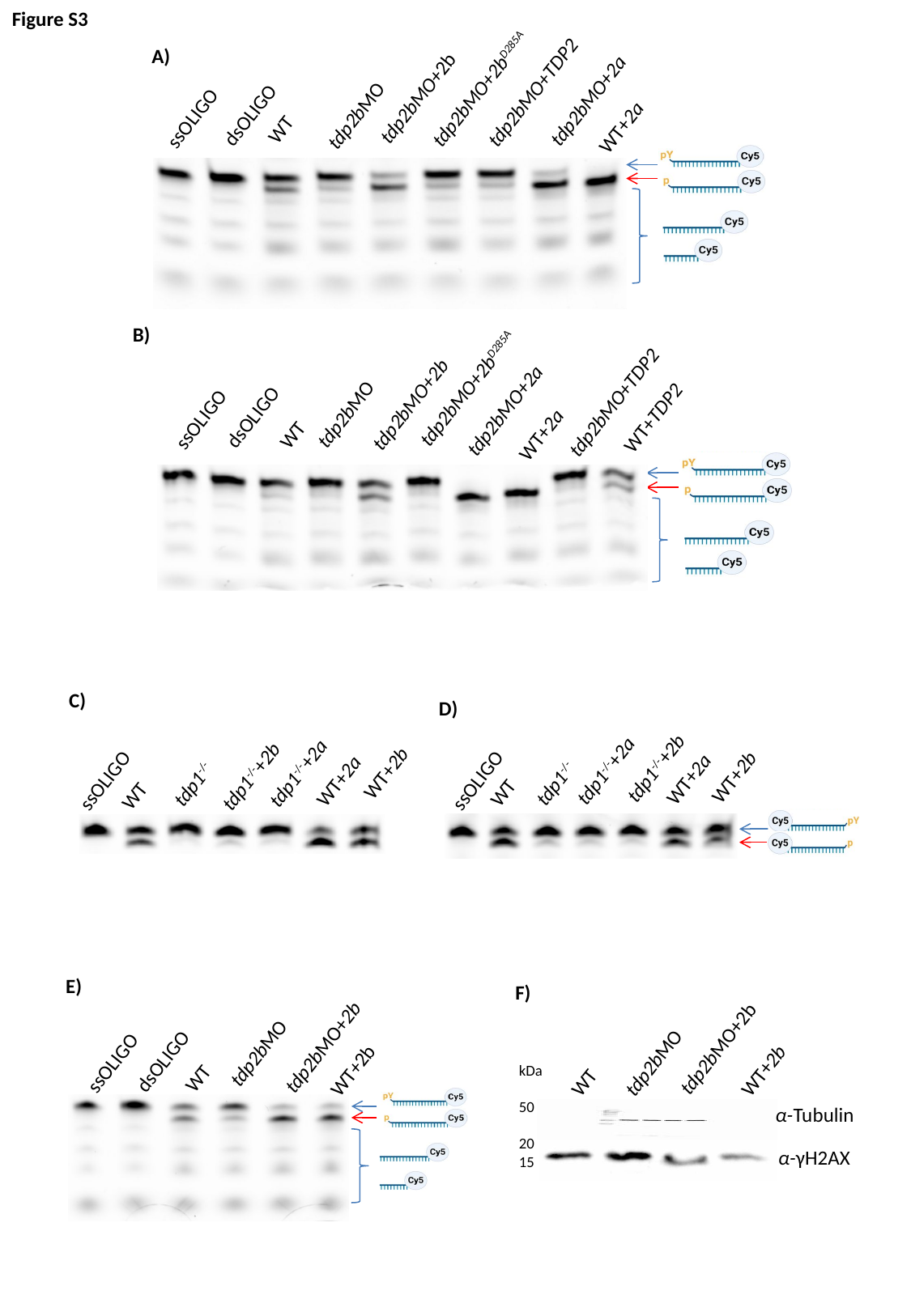

Figure S3
tdp2bMO+2a
ssOLIGO
dsOLIGO
tdp2bMO
WT
A)
tdp2bMO+2b
tdp2bMO+2bD285A
tdp2bMO+TDP2
WT+2a
tdp2bMO+2a
tdp2bMO
WT
dsOLIGO
ssOLIGO
B)
tdp2bMO+2b
tdp2bMO+2bD285A
tdp2bMO+TDP2
WT+2a
WT+TDP2
tdp1-/-+2b
ssOLIGO
WT
C)
tdp1-/-+2a
WT+2b
WT+2a
tdp1-/-
tdp1-/-+2b
ssOLIGO
WT
D)
tdp1-/-+2a
WT+2b
WT+2a
tdp1-/-
tdp2bMO+2b
tdp2bMO
dsOLIGO
ssOLIGO
WT+2b
WT
E)
kDa
50
20
15
tdp2bMO+2b
WT
WT+2b
tdp2bMO
α-Tubulin
α-γH2AX
F)

## Slide 4
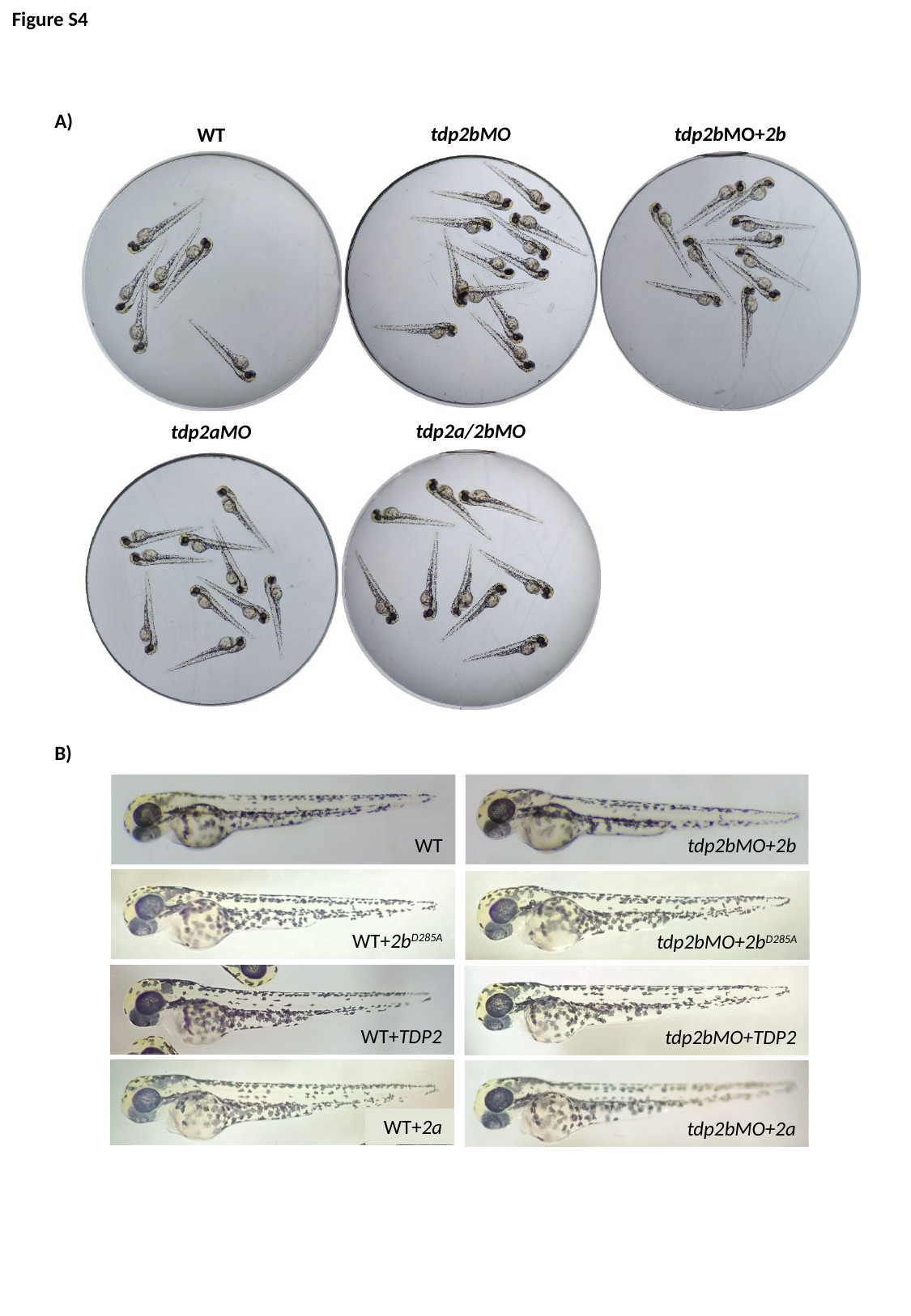

Figure S4
A)
tdp2bMO
tdp2bMO+2b
WT
tdp2a/2bMO
tdp2aMO
B)
WT
tdp2bMO+2b
WT+2bD285A
tdp2bMO+2bD285A
WT+TDP2
tdp2bMO+TDP2
WT+2a
tdp2bMO+2a

## Slide 5
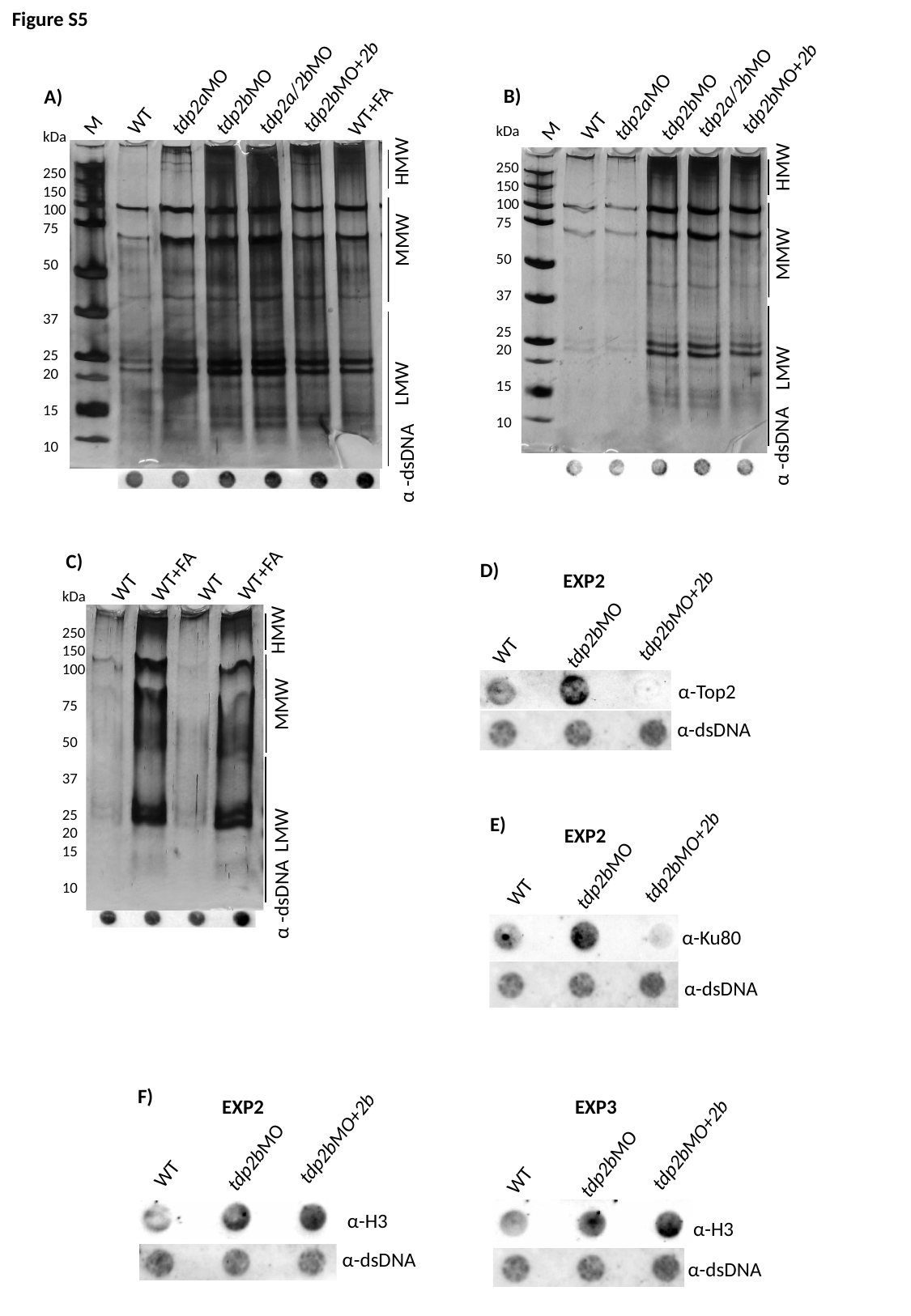

Figure S5
tdp2a/2bMO
WT
tdp2bMO
M
kDa
250
150
100
75
50
37
25
20
15
10
HMW
MMW
LMW
α -dsDNA
tdp2bMO+2b
WT+FA
tdp2aMO
A)
tdp2a/2bMO
WT
tdp2bMO
M
kDa
250
150
100
75
50
37
25
20
15
10
HMW
MMW
LMW
α -dsDNA
tdp2bMO+2b
tdp2aMO
B)
WT+FA
WT
WT+FA
WT
kDa
250
150
100
75
50
37
25
20
15
10
HMW
MMW
LMW
α -dsDNA
C)
D)
tdp2bMO+2b
WT
tdp2bMO
α-Top2
α-dsDNA
EXP2
tdp2bMO+2b
WT
tdp2bMO
α-Ku80
α-dsDNA
E)
EXP2
F)
tdp2bMO+2b
WT
tdp2bMO
α-H3
α-dsDNA
EXP2
tdp2bMO+2b
WT
tdp2bMO
α-H3
α-dsDNA
EXP3

## Slide 6
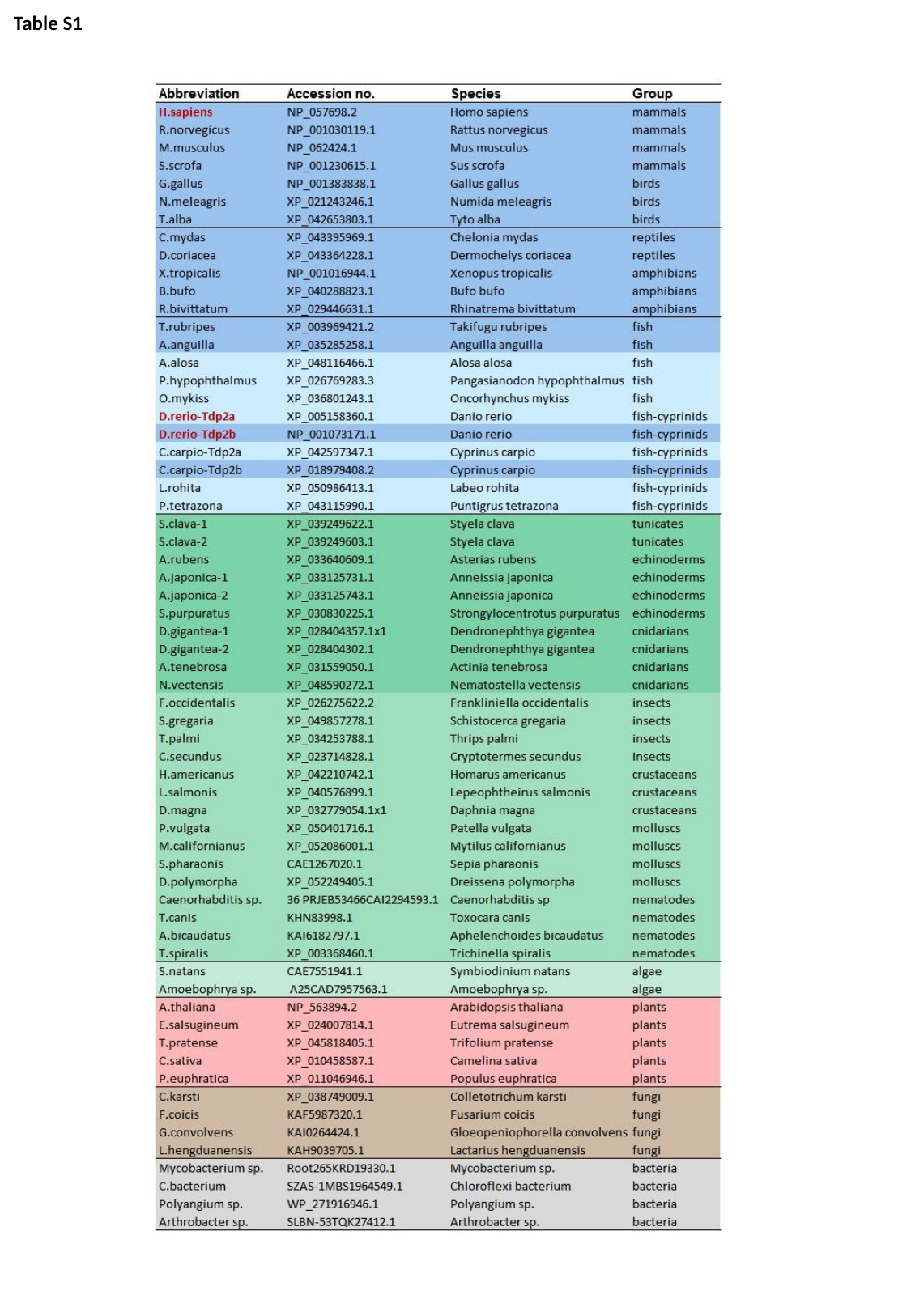

Table S1
